# Oxidative stress-induced DNA damage and apoptosis in multiple cancer cell lines: novel anticancer properties of marine *Aspergillus oryzae* NGM91 extract†

**DOI:** 10.1039/d5ra02028j

**Published:** 2025-05-22

**Authors:** Rana Hussein Naser, Zahraa Falah Azeez, Zinab Alatawi, Amani Albalawi, Taghreed Shamrani, Aisha M. A. Shahlol, Mohammad EL-Nablaway, Hanadi A. Alahmadi, Ghfren S. Aloraini, Nagwa A. Tharwat, Amr Fouda, Ahmed Ghareeb

**Affiliations:** a Department of Science, College of Basic Education, University of Diyala 32001 Baqubah Iraq ranaalqaysi@uodiyala.edu.iq; b College of Biotechnology, University of Al-Qadisiyah Iraq zahraa.azeez@qu.edu.iq; c Department of Family and Community Medicine, Faculty of Medicine, University of Tabuk Tabuk 47512 Saudi Arabia zalatawi@ut.edu.sa; d Family Medicine Department, King Salman Armed Forces Hospital Tabuk Saudi Arabia aalbalawi10@nwafh.med.sa; e Department of Clinical Biochemistry, Faculty of Medicine, King Abdulaziz University Jeddah Saudi Arabia tshumrani@kau.edu.sa; f Food, Nutrition and Lifestyle Unit, King Fahd Medical Research Centre, King Abdulaziz University Jeddah Saudi Arabia; g Medical Lab Technology Department, Faculty of Medical Technology, Wadi-Al-Shatii University Brack Libya as.shahlol@wau.edu.ly; h Department of Basic Medical Sciences, College of Medicine, AlMaarefa University P. O. Box 71666 Riyadh 11597 Saudi Arabia mnablawi@um.edu.sa; i Department of Medical Biochemistry, Faculty of Medicine, Mansoura University Mansoura 35516 Egypt medo_bio@mans.edu.eg; j College of Health Science and Nursing, Al-Rayan National Colleges Madinah 42541 Saudi Arabia ha.alahmadi@amc.edu.sa; k Department of Medical Laboratory, College of Applied Medical Sciences, Prince Sattam Bin Abdulaziz University Al-Kharj 11942 Saudi Arabia g.aloraini@psau.edu.sa; l Department of Botany and Microbiology, Faculty of Science, Cairo University Giza 12613 Egypt nagwa@sci.cu.edu.eg; m Botany and Microbiology Department, Faculty of Science, Al-Azhar University Nasr City Cairo 11884 Egypt amr_fh83@azhar.edu.eg; n Botany and Microbiology Department, Faculty of Science, Suez Canal University Ismailia 41522 Egypt aghareeb@science.suez.edu.eg

## Abstract

This study explored the metabolites and bioactive potential of the ethyl acetate extract from a marine-derived fungal strain, *Aspergillus oryzae* NGM91, isolated from Red Sea sediments. Chemical profiling through FT-IR, GC-MS, and HPLC analysis revealed a complex composition dominated by benzyl benzoate (79.99%) and rosmarinic acid (162.15 μg ml^−1^) as major constituents. The fungal extract exhibited potent free radical scavenging activity (DPPH IC_50_ = 17.26; ABTS IC_50_ = 27.91 μg ml^−1^), with high total antioxidant capacity (476.57 μg per mg AAE) and ferric reducing power (302.62 μg per mg AAE). It demonstrated selective COX inhibition (COX-1 IC_50_: 20.66 μg ml^−1^; COX-2 IC_50_: 36.32 μg ml^−1^). Cytotoxic screening showed significant activity against PC3 cells (IC_50_: 70.47 μg ml^−1^), PANC-1 (IC_50_: 90.42 μg ml^−1^), HepG2 (IC_50_: 100.36 μg ml^−1^), and Caco-2 cells (IC_50_: 104.69 μg ml^−1^), while exhibiting lower cytotoxicity towards normal Wi-38 fibroblasts (IC_50_: 230.31 μg ml^−1^). In PC3 cells, the extract induced oxidative stress markers (MDA: 13.63 μmol mg^−1^; NO: 66.13 μmole per mg), modulated antioxidant enzyme activities, upregulated antioxidant genes (CAT: 1195%, SOD: 788%, GPx: 473%, GST: 251%), increased DNA damage (205.1%), activated apoptotic pathways *via* BCL-2 downregulation and BAX/P53/Caspase-3 upregulation, and induced G1 phase arrest (72.97%). These findings demonstrate *A. oryzae* NGM91's therapeutic potential through oxidative stress-mediated DNA damage and apoptotic cell death induction in PC3 cancerous cells.

## Abstract 2

This study explored the metabolites and bioactive potential of the ethyl acetate extract from a marine-derived fungal strain, *Aspergillus oryzae* NGM91, isolated from Red Sea sediments.

## Graphical abstract

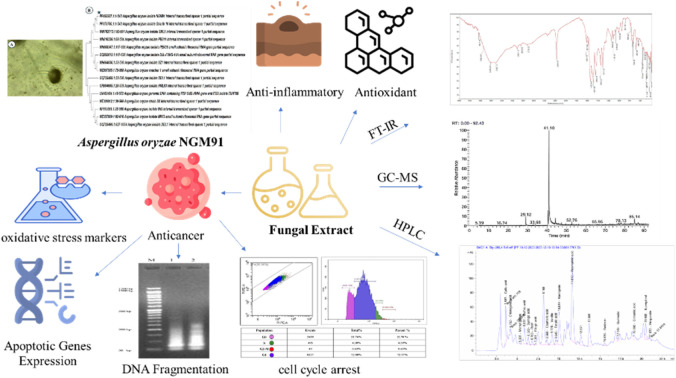

## 1 Introduction

In 2020, the International Agency for Research on Cancer (IARC) reported a total of 18.1 million new cases of cancer worldwide. Out of these, 9.3 million cases were in men, and 8.8 million were in women. In addition to the 18.1 million new cases in 2020, cancer was the leading cause of death globally that year, resulting in about 10 million deaths. The World Health Organization (WHO) estimates that there will be more than 28 million new cases of cancer in 2040, and there may be more than 16 million cancer deaths worldwide in that year.^1^ Cancer brings forth extensive economic and social challenges for developing countries while simultaneously hindering the worldwide progress of the healthcare system.^2^

Oncologists commonly use surgery, radiation, and chemotherapy as their primary approaches in cancer treatment, and chemotherapy is the most common alongside surgery.^3^ Nevertheless, the constant administration of drug-based cancer treatment can impair the patient's immune system.^4^ Furthermore, the increasing ability of cancer cells to become resistant to currently available anti-cancer drugs poses a serious concern.^5^ As a result, natural products have become recognized as a potential alternative source in the search for new, potent, and pharmaceutically active anti-cancer agents.^6^ Most of the anti-cancer drugs available commercially today originate from natural compounds or synthetic versions.^7^ Some well-known anti-cancer medications like camptothecin, podophyllotoxin, vinblastine, vincristine, and Taxol are derived from natural products and continue to be isolated from their plant sources.^8^ However, the cultivation, procurement, and authentication of plant materials for natural product drug discovery involve intensive labor inputs, considerable time investments, and substantial ecological and financial costs.^7^

Prostate cancer prevalence has grown markedly worldwide, with age-standardized incidence rates climbing 13.16% between 1990–2019. The Eastern Mediterranean saw the steepest increase at 72.43%, while North Africa and the Middle East regions experienced 77% higher incidence and 144% greater prevalence during this period, despite unchanged mortality rates.^9,10^ This expanding health challenge is further complicated by tumors frequently developing resistance to both androgen-deprivation therapy (ADH) and chemotherapy treatments.^11^ Fungal metabolites show significant promise against prostate cancer, particularly the treatment of castration-resistant prostate cancer (CRPC).^12^ Recent research has identified several metabolites with potent anticancer properties, for example, acetyl aszonalenin, derived from *Aspergillus neoniveus*, effectively blocks proliferation and migration across numerous prostate cancer cells, notably in CRPC variants. This compound works through cannabinoid receptor engagement, revealing a distinctive therapeutic pathway.^13^*Penicillium dimorphosporum* produces deoxy-14,15-dehydroisoaustamide, which tackles drug resistance by demolishing specific androgen receptor variants, renewing prostate cancer susceptibility to enzalutamide treatments. Deoxy-14,15-dehydroisoaustamide from fungus *Penicillium dimorphosporum* breaks down resistance-linked androgen receptor variants, reactivating enzalutamide's effectiveness (androgen receptor blockers) against resistant prostate tumors.^14^

Fungi are eukaryotic microorganisms and macroorganisms that belong to the Kingdom Mycota. They have been a part of human existence for millennia. Ancient civilizations utilized fungi for a variety of functions including as a food source, to produce alcoholic drinks, for medicinal purposes, and for cultural practices.^15^ The fungal kingdom is estimated to have between 2.2 to 3.8 million diverse species. Of these, about 120 000 species have been scientifically accepted and classified. Furthermore, new fungal species are continually being uncovered in various environments like aquatic habitats, tropical forest flora, and soil ecosystems associated with insects.^16^ Fungi collected from various habitats have been thoroughly studied for their elaborate secondary metabolites. These metabolites exhibit great structural variety and unique chemical and biological activities.^17^

The fungal genera *Aspergillus* and *Penicillium* account for around one-third of all fungal metabolites discovered. *Aspergillus* is a ubiquitous, fast-growing fungus with approximately 378 known species, 180 of which are of pharmaceutical and commercial value according to the World Data Centre for Microorganisms (WDCM).^18,19^ Compared to other fungal genera, the secondary metabolites extracted from *Aspergillus* display particularly diverse and fascinating biological activities like antimicrobial, antioxidant, cytotoxic, and antiviral effects.^20,21^ Given its immense diversity, *Aspergillus* remains one of the most prolific and important sources of novel secondary metabolites exhibiting anti-inflammatory, anticancer, antioxidant and antibacterial properties. Its abundance and metabolic variety cement *Aspergillus* as a prime target for investigations aimed at discovering new bioactive compounds.^22,23^ For example, investigations into the fungus *Aspergillus oryzae* revealed its ability to generate l-glutaminase, an amidohydrolase enzyme that breaks down l-glutamine into l-glutamic acid and ammonia, recognized for its role in lymphoblastic leukemia. The isolated strain demonstrated notable enzymatic productivity, reaching peak activity measurements of 217.65 IU, and exhibited anti-cancer properties against MCF-7 breast cancer cells, with a reported IC_50_ of 283.288 μg ml^−1^.^24^ Additionally, two fungal isolates – *Neosartorya hiratsukae* and *Neosartorya pseudofischeri* were obtained from soil samples and screened for activity against HepG2, MCF-7, L929, HeLa, HT-29, KB, Vero and P388 cancer cell lines, IC_50s_ values ranged from 144.31 to 267.73 μg ml^−1^.^25^ Another study on the fungus *Chaetomium globosum* led to the isolation of the compound methoxy-2,2-dimethyl-4-octa-4,6-dienyl-2*H*-naphthalene-1-one, which displayed potent anti-cancer effects against the A549, MCF-7, PC-3, and THP-1 cancer cell lines when tested in a dose-dependent manner with IC_50_ concentrations of 9.89–54 μg ml^−1^.^26^

Marine organisms inhabiting the Red Sea shores represent an untapped frontier for discovering novel bioactive compounds, with fungi particularly standing out as an understudied group. Our research focused on fungi collected from Red Sea coastal sediments, investigating their potential as sources of therapeutic agents. After isolating various fungal strains from these marine sediments, we conducted comprehensive screening that led to the identification of *Aspergillus oryzae* NGM91 as the most promising isolate. Extensive chemical profiling of the fungal extract was carried out using FT-IR, HPLC, and GC-MS. The research examined the extract's ability to neutralize free radicals and modulate inflammatory responses. Furthermore, we evaluated its selective cytotoxicity against multiple cancer cell lines while sparing normal cells. The study also elucidated the underlying cellular stress responses, particularly focusing on oxidative stress-induced DNA damage and apoptotic cell death in PC3 cancerous cells.

## 2 Materials and methods

### 2.1. Culture-dependent isolation of fungi and downstream enrichment of bioactive metabolites

750 ml of marine sediment specimens were procured from Saudi Arabia's western Red Sea shoreline during February 2024. The samples were collected using sterile flasks, then subsequently kept at 4 °C in thermal-controlled containers, and then transferred to laboratory premises. The collected samples were first diluted using 0.9% isotonic saline in preparation for microbial isolation. The diluted samples were then inoculated onto Potato Dextrose Agar plates made with seawater using the spread plate technique. After inoculation, the plates were incubated and checked periodically for fungal growth starting 14 days later. Over the incubation period, fungal colonies developed on the plates. The number of colonies on each plate was counted to calculate the frequency of fungi occurrence in the samples. In addition, the cultural characteristics of the colonies were examined through observation and microscopy to identify and enumerate the different fungal types.^27^

The fungal isolates were then grown in yeast extract broth by inoculating the broth and incubating for 14 days at 25 °C. After incubation, the fungal mycelium was separated from the surrounding supernatant by filtration using Whatman filter paper no. 1. The recovered supernatant underwent three sequential liquid–liquid extractions with ethyl acetate (1 : 1 v/v, 250 ml each), with 15 minutes of vigorous shaking per extraction cycle. This process efficiently transferred the bioactive compounds from the aqueous phase to the organic phase. Using liquid–liquid extraction to transfer compounds into the organic phase.^28^ The ethyl acetate fraction was pooled and concentrated by rotary evaporation to obtain the concentrated extract containing secreted fungal metabolites.

Fungal genomic DNA was extracted using DNeasy plant kit (Qiagen, CA, USA). PCR amplification was performed in a 50 μl reaction containing 25 μl Master Mix, 3 μl each of ITS-1 and ITS-4 primers (10 pmol μl^−1^), 3 μl template DNA (10 ng μl^−1^), and 16 μl dH_2_O. Amplicons were purified and sequenced *via* Sanger Sequencing on a 3500 Series Genetic Analyzer (Applied Biosystems, USA). 18S rRNA gene sequences were analyzed using BLAST, deposited in GenBank, and a neighbor-joining phylogenetic tree was constructed using MEGA7 software.^29^

### 2.2. Chemical analysis of the fungal crude extract

#### 2.2.1. FT-IR spectroscopy

The functional groups of the ethyl acetate extract of *Aspergillus oryzae* NGM91 were analyzed by FTIR spectroscopy. The extract was dried in a thermostatted desiccator at 45 °C for 24 hours and blended thoroughly with KBr. A thin disc was prepared from the extract–KBr mixture and analyzed on an FTIR spectrometer (Nicolet 6700, Thermo Fisher Scientific, USA)^30^ in the range of 400–4000 cm^−1^. This allowed the detection of the extract's functional groups based on characteristic absorption bands.

#### 2.2.2. GC-mass spectrometry analysis

The chemical composition of the fungal extract was analyzed using a TRACE GC1310-ISQ mass spectrometer (Thermo Scientific, USA) equipped with a TG-5MS capillary column (30 m × 0.25 mm × 0.25 μm film thickness). The oven temperature was initially 35 °C, then increased by 3 °C min^−1^ to 200 °C, held for 3 min, further increased to 280 °C at 3 °C min^−1^, and held for 10 min. The GC system maintained the injector at 250 °C and the MS transfer line at 260 °C. Helium served as carrier gas (1 ml min^−1^). After 3 min solvent delay, the Autosampler AS1300 injected 1 μl samples in split mode. EI mass spectra acquisition occurred at 70 eV across *m*/*z* 40–1000 range (full scan), with the ion source heated to 200 °C.^31^ Component identification utilized retention time comparison and mass spectral matching against Wiley 09 and NIST 11 libraries.

#### 2.2.3. HPLC analysis

The analysis utilized an Agilent 1260 HPLC system (Agilent Technologies, USA) fitted with Zorbax Eclipse Plus C8 column (4.6 × 250 mm, 5 μm). Mobile phases consisted of water (A) and acetonitrile with 0.05% trifluoroacetic acid (B). Gradient protocol proceeded: 0–1 min (82% A), 1–11 min (75% A), 11–18 min (60% A), 18–22 min (82% A), 22–24 min (82% A). Flow rate remained 0.9 ml min^−1^ for the entire 24 min run. Detection occurred at 280 nm using variable wavelength detector. Samples underwent injection at 5 μl volume. The column temperature stayed at 40 °C throughout analysis.^32^

### 2.3. Evaluating the free radical scavenging activity of fungal extract

#### 2.3.1. DPPH antioxidant screening

A 0.1 mM DPPH solution in ethanol was prepared. 1 ml of this solution was added to 3 ml of fungal extract solutions at varying concentrations (3.9–1000 μg ml^−1^) prepared by diluting ethanol-soluble extracts. The mixtures were shaken and incubated at room temperature for 30 minutes. Absorbance was then measured at 517 nm using (UV-Vis Milton Roy, USA) spectrophotometer.^33^DPPH scavenging% = (*A*_0_ − *A*_1_)/*A*_0_ × 100where *A*_0_ is the absorbance of control and *A*_1_ is the absorbance of the extract sample.

#### 2.3.2. ABTS˙^+^scavenging test

The ABTS radical cation (ABTS˙^+^) was generated by reacting 7 mM ABTS stock solution with 2.45 mM K_2_S_2_O_8_, allowing this mixture to stand at room temperature in the dark. The assay mixture was prepared by adding 0.07 ml of the fungal extract sample to 3 ml of diluted ABTS˙^+^ solution. After incubating for 6 minutes, the absorbance was measured at 734 nm using a spectrophotometer.^34^ The radical scavenging capacity was expressed relative to the standard antioxidant gallic acid which served as the reference.ABTS˙^+^ inhibition% = (Ac − As)/Ac × 100

#### 2.3.3. TAC evaluation

The phosphomolybdenum assay based on spectrophotometric analysis evaluated the fungal extract's total antioxidant capacity. The fungal extract sample was incubated with the phosphomolybdenum reagent, and the absorbance at 630 nm was measured using a Biotek ELX800 microplate reader (BioTek Instruments, USA).^35^ The total antioxidant capacity was expressed as ascorbic acid equivalents (AAE) in μg mg^−1^ of fungal extract.

#### 2.3.4. FRAP quantification

FRAP testing of the fungal extract was determined by the K_3_[Fe_6_] reduction method. The fungal extract sample was incubated with K_3_[Fe(CN)_6_] and CCl_3_COOH, and the absorbance was measured at 630 nm using a Biotek ELX800 microplate reader (BioTek Instruments, USA). DMSO was employed as the negative control, and 1 mg per ml ascorbic acid was used as the positive control.^36^ Based on an ascorbic acid standard curve, the reducing power was quantified as ascorbic acid equivalents (AAE) in μg mg^−1^ of fungal extract sample.

### 2.4. Fungal extract: COX-1/2 inhibitory anti-inflammatory assessment

The extract from fungi was solubilized in DMSO and evaluated at twelve concentration levels (0.5–1000 μg ml^−1^) within 1 ml final volume, with tests conducted in triplicate. A microplate reader was used to measure the inhibitory activity and identify the increase in absorbance at 611 nm (BIOTEK; USA).^37,38^ IC_50_ was calculated from the graph, and celecoxib was used as a positive control for the COX-2 inhibition assay.Cox-inhibition% = (1 − As/Ac) × 100

### 2.5. Assessing the cytotoxicity and anti-cancer activity of the fungal extract

RPMI-1640 medium containing 10% FBS, 1% penicillin/streptomycin maintained cells at 37 °C (5% CO_2_) (Thermo Fisher Scientific, 11875093). Test compounds (10 mg ml^−1^) dissolved in DMSO (lipophilic extracts) or sterile water (hydrophilic extracts), filtered through a 0.22 μm membrane, and stored at −20 °C. Fresh dilutions were prepared in 2% serum RPMI before each experiment, keeping DMSO below 0.5% to prevent solvent toxicity effects. Cells are passaged every 3–4 days at 80% confluence using trypsin–EDTA (0.25%).^39^

A 96-well tissue culture plate was seeded with 100 μl per well containing 1 × 10^5^ cells per ml of WI38 normal human lung fibroblasts and incubated at 37 °C for 24 hours to allow formation of a complete cell monolayer sheet. The cytotoxicity of fungal extract was first tested against the WI38 cells to determine its toxicity. The fungal extract was then tested as an anti-cancer agent against four cancerous cell lines: Caco-2 colon cancer cells, PC-3 prostate cancer cells, PANC-1 pancreatic cancer cells, and HepG2 hepatocellular carcinoma cells. The cancer cell lines were acquired from VACSERA (Holding Company for Biological Products and Vaccines) located in Cairo, Egypt. Serial two-fold dilutions of fungal extract were added to wells containing confluent cell monolayers that had been washed twice after growth medium removal. The preparation used RPMI medium containing 2% serum (maintenance medium), with three wells reserved as controls containing only maintenance medium. Plates underwent incubation at 37 °C while being monitored for cytotoxicity signs. An MTT solution at 5 mg ml^−1^ in PBS was added to each well, shaken at 150 rpm for 5 minutes, and incubated at 37 °C and 5% CO_2_ for 4 hours to allow MTT metabolism. The media was discarded, the plate dried, and the formazan resuspended in DMSO by shaking at 150 rpm for 5 minutes and the optical density was measured at 560 nm.^40^

### 2.6. Evaluation of oxidative stress damage in cancerous cell lines

#### 2.6.1. Malondialdehyde (MDA)

4.0 mM TBA (57.66 mg/100 ml) was prepared daily in glacial acetic acid. MDA standards (0.1–1.0 μM) were created from 1 mM stock solution (TBA in glacial acetic acid) (31.35 mg/100 ml). From the stock solution, different concentrations of 0.1, 0.2, 0.4, 0.8 and 1.0 μM were prepared. The fungal extract was prepared by dissolving in C_2_H_5_OH at desired concentration. Equal volumes (1 ml) of TBA were mixed with either MDA standards, fungal extract, or blank. The mixtures were heated at 95 °C for 30 minutes, cooled to room temperature, then absorbance was measured at 534 nm. The sample readings were taken against blank while standards were measured at 534 nm against distilled water using spectrophotometer (UV-Vis Milton Roy, USA).^41^

#### 2.6.2. Nitric oxide (NO)

NO was measured *via* nitrite (NO_2_^−^) determination using Biodiagnostic company kits (STA-802, Egypt). The assay mixture contained NaNO_2_, 50 μmol L^−1^, and diazotizing reagent (NH_2_C_6_H_4_SO_2_NH_2_, 10 mmol L^−1^). The fungal extract (0.1 ml) was combined with sulphanilamide solution and incubated for 5 minutes. *N*-(1-Naphthyl) ethylenediamine (NEDA, C_10_H_7_NHCH_2_CH_2_NH_2_, 1 mmol L^−1^) was then added as a coupling reagent, forming a pink-purple azo compound. After 5 minutes, the absorbance was measured at 540 nm against the sample blank.^42^

The nitrite concentration in μmole L^−1^ was calculated as: (absorbance of fungal extract/absorbance standard) × 50.

#### 2.6.3. Catalase (CAT)

CAT activity was determined by spectrophotometric measurement of H_2_O_2_ decomposition at 240 nm at room temperature. The reaction mixture (3.0 ml total) contained 100 mM sodium phosphate buffer (pH 7.0), 30 mM H_2_O_2_, and 100 μl fungal extract. One enzyme unit was defined as 0.001 absorbance change per minute under these experimental conditions.^43^ The absorbance decrease was monitored to evaluate the fungal extract's catalase activity.

#### 2.6.4. Glutathione peroxidase (GPx)

GPx was measured using Biodiagnostic company kits (Cayman Chemical, 703102, USA). The fungal extract was added to a reaction mixture containing glutathione, glutathione reductase, and NADPH. Hydrogen peroxide initiated the enzyme reaction. The absorbance decrease at 340 nm was monitored spectrophotometrically (extinction coefficient: 6220 M^−1^ cm^−1^).^44^ GPx activity in the fungal extract was determined from the decline in rate of absorbance.

#### 2.6.5. Glutathione reduced

The total glutathione of the fungal extract was measured in a reaction mixture (1 ml) containing NADPH (100 μl, 2.1 mM), DTNB (100 μl, 6 mM), glutathione reductase (20 μl), and fungal extract (200 μl) in sodium phosphate buffer (125 mM, pH 7.5) with EDTA (6.3 mM). The optical density changes were monitored at 412 nm and 25 °C.^45^

#### 2.6.6. Superoxide dismutase (SOD)

SOD activity was evaluated through nitroblue tetrazolium (NBT) photoreduction inhibition assay. The reaction mixture (3.0 ml) contained 50 mM Na_2_HPO_4_ (pH 7.6), 0.1 mM C_10_H_16_N_2_O_8_, 50 mM Na_2_CO_3_, 12 mM l-methionine, 50 μM NBT, 10 μM riboflavin, and 100 μl fungal extract. The mixture was exposed to white light for 15 minutes at room temperature, followed by absorbance measurement at 560 nm. One enzyme unit represented the amount of fungal extract causing 50% inhibition of NBT photochemical reduction. A control reaction without fungal extract was run parallel.^46^

### 2.7. Apoptotic and antioxidant-related genes expression

Total RNA was isolated from control and treated PC3 prostate cancer cells using the RNeasy Mini Kit (Qiagen, Hilden, Germany) with DNaseI digestion. The RNA was further purified using RQ1 RNAse-free DNAse (Invitrogen, Germany) and then resuspended in DEPC-treated water. RNA concentration was measured at 260 nm, with purity confirmed by 260/280 nm ratios of 1.8–2.1.^47^

qRT-PCR was performed using StepOne™ System (Applied Biosystems, USA). Each 25 μl reaction contained 12.5 μl SYBR Premix Ex Taq (TaKaRa), 0.5 μl each of sense and antisense primers (0.2 mM), 5 μl cDNA template, and 6.5 μl distilled water. Primers for antioxidant genes (CAT, SOD, GPx, GST) and apoptotic genes (Bcl-2, BAX, P53, Caspase-3)^48,49^ were designed as listed in Table S1.† Melting curve analysis at 95 °C verified primer quality. Gene expression was calculated using 2^*−*ΔΔCT^ method.^50^

### 2.8. Assessment of DNA strand breakage *via* COMET assay

The cell preparation involved tryptic digestion to obtain single-cell preparations, followed by embedding 1.5 × 10^4^ cells within 0.75% low-gelling agarose matrices on pre-treated microscope slides. Cell lysis proceeded at 50 °C for 4 hours using a buffer containing 0.5% SDS and 30 mM EDTA at pH 8.0. The samples underwent overnight equilibration in TBE buffer before subjection to electrophoretic separation (0.6 V cm^−1^, 25 minutes).^51^ Nuclear material visualization relied on propidium iodide staining, with subsequent fluorescence microscopy imaging. The classification framework encompassed intact DNA without visible migration (class 0), minimal migration less than the nuclear diameter (class 1), intermediate damage with tails extending 1–2× nuclear width (class 2), and severe fragmentation displaying migration beyond twice the nuclear diameter (class 3).^52^

### 2.9. Analysis of DNA fragmentation pattern

#### 2.9.1. DNA gel electrophoresis laddering assay

The cancer cell line (1 × 10^6^) was centrifuged (800 rpm, 10 min) in 1 ml medium. Cells underwent lysis (30 min, on ice) in buffer [10 mM Tris (pH 7.4), 150 mM NaCl, 5 mM C_10_H_16_N_2_O_8_, 0.5% Triton X-100]. After vortexing and centrifugation (10 000 g, 20 min), DNA was extracted using C_6_H_5_OH : CHCl_3_ : C_5_H_12_O (25 : 24 : 1) mixture. Samples were analyzed on 2% agarose containing 0.1 μg per ml C_21_H_20_BrN_3_.^53^

#### 2.9.2. Colorimetric DNA analysis *via* diphenylamine method

The cancer-selected cells were lysed in 0.5 ml buffer [10 mM Tris–HCl (pH 8), 1 mM EDTA, 0.2% Triton X-100] and centrifuged (10 000 rpm, 20 min, 4 °C). Both pellets and supernatants received 25% TCA, incubated (24 h, 4 °C), then centrifuged. Pellets were resuspended in 5% TCA (83 °C, 20 min). DPA solution [150 mg DPA, 10 ml glacial acetic acid, 150 μl H_2_SO_4_, 50 μl acetaldehyde (16 mg ml^−1^)] was added and incubated (24 h, RT).^54^

DNA fragmentation was calculated using 600 nm absorbance readings.% fragmented DNA = [OD(S)/(OD(S) + OD(P))] × 100OD represents optical density measurements of supernatant (S) and pellet (P) fractions.

### 2.10. Cell cycle distribution analysis of PC3 cells

PC3 cells (1 × 10^6^/ml) were seeded in 6-well plates and treated with fungal metabolites (0.25, 2.5, 50 ppm) or left untreated as a control. After 48 h and 72 h incubation (37 °C, 5% CO_2_), cells were trypsinized, centrifuged (400 g, 5 min), and PBS-washed. Pellets were resuspended in 250 μL trypsin buffer, vortexed, mixed with 200 μL tyrosine inhibitor/RNase buffer, and incubated 10 min. Cold PI solution (200 μL) was added for 10 min at 4 °C in darkness with stirring. CytoFlex (Beckman Coulter,USA)^55^ was used for acquisition, and CytExpert software for analysis.

### 2.11. Statistical analysis

Statistical analyses were conducted using SPSS software version 22 (USA) using the Shapiro–Wilk test to test the data's normality. One-way analysis of variance (ANOVA) was employed to assess differences between experimental groups, followed by *post hoc* comparisons (Tukey's HSD) to identify specific group differences.

## 3 Results and discussion

### 3.1. Isolation and molecular classification of the fungal isolate

**Fig. 1:**
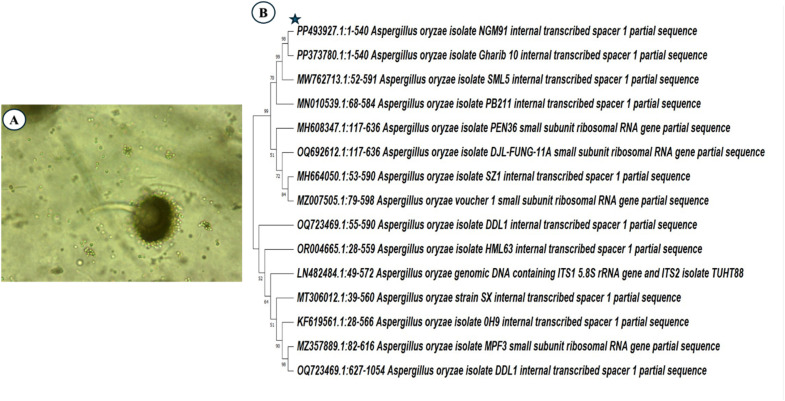
(A) Microscopic examination of *Aspergillus oryzae* NGM91 (B) phylogenetic association of isolate NGM91 with closely related *Aspergillus* species.

Eight fungal isolates were collected from the Red Sea sediments using sequential dilution techniques. Through cross-streak screening, isolate NGM91 was identified as the most promising strain among the collected isolates. Microscopic examination of the isolate revealed characteristic morphological features of *Aspergillus oryzae*, displaying the distinctive reproductive structures of *Aspergillus* sp. The microscopy image shows a prominent dark spherical vesicle with radiating conidial chains surrounding its surface. The conidiophore appears unbranched, extending from the vesicle base, which is typical of *Aspergillus* species. Multiple small, spherical conidia are visible scattered in the surrounding field, exhibiting a greenish coloration characteristic of *A. oryzae* (Fig. 1a).

Molecular identification through sequence analysis (accession number PP493927) confirmed the morphological findings, placing the isolate NGM91 within a well-supported *A. oryzae* clade in the phylogenetic tree. The phylogenetic analysis demonstrates particularly close evolutionary relationships with *A. oryzae* isolate Gharib 10 (PP373780) and isolate SML5 (MW762713), as evidenced by their high bootstrap value of 98–99%, suggesting very recent common ancestry (Fig. 1b).

### 3.2. Chemical profiling of the fungal metabolites

#### 3.2.1. FT-IR

**Fig. 2:**
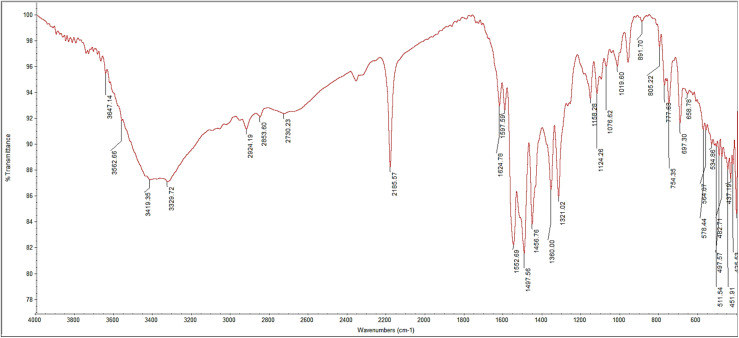
FT-IR spectrum of crude extract of *Aspergillus oryzae* NGM91.

The FT-IR spectrum of crude extract from *Aspergillus oryzae* NGM91 using an ethyl acetate liquid–liquid extraction technique showed a broad O–H stretch at 3423 cm^−1^ indicating hydrogen bonded alcohols or phenols, a strong alkane C–H stretch at 2927 cm^−1^ revealing alkyl groups, a carbonyl C=O stretch at 1647 cm^−1^ characteristic of ketones or aldehydes, a N–H bend at 1541 cm^−1^ and C–N stretch at 1242 cm^−1^ signifying amines, and a strong C–O stretch at 1078 cm^−1^ typical of alcohols or ethers (Fig. 2) (Table 1).

**Table 1:** FT-IR spectroscopic analysis of *A. oryzae* NGM91 extract

Observed peak wavelength (cm^−1^)	Functional group	Bond type	Characteristic absorption range (cm^−1^)	Relative intensity
3423	O–H stretch	Hydrogen bond	3200–3600	Strong, broad
2927	C–H stretch	Alkane	2850–3000	Strong
1647	C=O stretch	Carbonyl	1690–1760	Medium
1541	N–H bend	Amine	1500–1600	Medium
1242	C–N stretch	Amine	1210–1360	Strong
1078	C–O stretch	Alcohol	1000–1300	Strong

#### 3.2.2. GC-MS analysis

The GC-MS chromatogram and accompanying data table provided a comprehensive analysis of a complex chemical mixture. The chromatogram displayed numerous peaks spanning a wide retention time range from around 5 to over 85 minutes, indicating the presence of a diverse array of compounds.

**Fig. 3:**
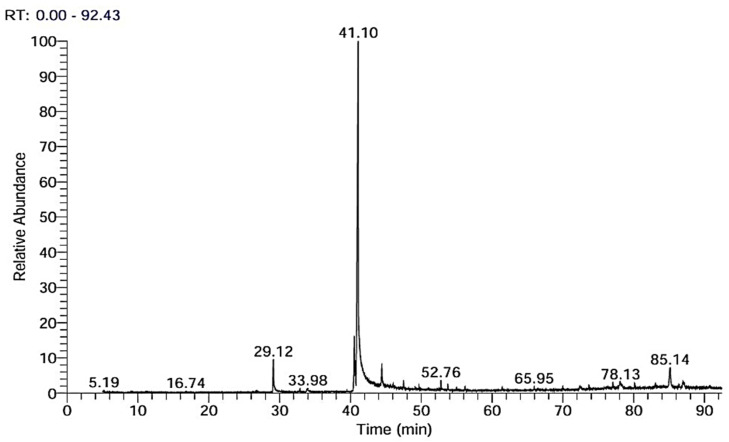
GC-MS chromatogram of crude extract from *Aspergillus oryzae* NGM91 revealing the presence of numerous compounds including benzyl benzoate as the major constituent.

The tallest peak at 41.09 minutes corresponded to phenylmethyl benzoate (benzyl benzoate), which dominated the sample with an overwhelming 79.99% area percentage. Other significant components included 3-ethoxy-4-hydroxybenzaldehyde(ethyl vanillin) (29.11 minutes, 4.38% area), (2*E*)-3,7,11,15-tetramethylhexadec-2-en-1-yl(9*Z*,12*Z*)-octadeca-9,12-dienoate (phytyl linoleate) (85.14 minutes, 3.61% area), and 5-amino-2-(4-methoxyphenyl)-2-methyl-2*H*-[1,2,4]triazolo[1,5-*a*][1,3,5]triazine(44.41 minutes, 2.90% area). Interestingly, the analysis identified several minor constituents with area percentages below 1%, such as 2-ethoxyphenol (16.73 minutes, 0.11%), 2-*tert*-butyl-4-methoxyphenol (32.71 minutes, 0.05%), and (3β,5*Z*)-3,17-dihydroxypregn-5-en-20-one (52.96 minutes, 0.15%) (Fig. 3) (Table 2).

**Table 2:** Identified compounds with corresponding data from GC-MS analysis of *Aspergillus oryzae* crude extract

RT	Compound name	Chemical formula	MW	Area%
5.18	*N*-Ethylhydroxylamine	C_2_H_7_NO	61	0.15
5.27	2-(Methylamino)-2-methylpropanoic acid	C_5_H_11_NO_2_	117	0.03
5.39	1-Ethoxy-1,3,3-trimethoxypropane	C_8_H_18_O_4_	150	0.03
5.69	1-Acetyloxypropan-2-one	C_5_H_8_O_3_	146	0.07
5.76	2-(1-Methylpropyl)-2-methylpropane-1,3-diyl dicarbamate	C_10_H_20_N_2_O_4_	232	0.03
5.98	Cyclopent-4-ene-1,3-dione	C_5_H_4_O_2_	96	0.16
6.44	*Trans*-cyclopentane-1,2-diol	C_5_H_10_O_2_	102	0.09
7.08	5-*O*-Methyl-*N*,*N*-dimethyl-d-gluconamide	C_9_H_19_NO_6_	237	0.08
7.42	Ethyl (1,2,4-triazol-1-ylmethyl) carbonate	C_6_H_9_N_3_O_3_	171	0.05
8.64	4-Methyl-6-phenyl-1,3-oxazinane-2-thione	C_11_H_13_NOS	207	0.03
9.02	Prop-2-en-1-ol	C_3_H_6_O	58	0.16
11.13	2-[(7-Chloro-4-quinolyl)amino]benzoic acid 2-(dimethylamino)ethyl ester	C_19_H_17_CIN_2_O_4_	372	0.07
16.73	2-Ethoxyphenol	C_8_H_10_O_2_	138	0.11
26.3	2-(*N*,*N*,*N*-Trimethylhydrazino)-1,3-benzothiazole	C_10_H_13_N_3_S	207	0.12
29.11	3-Ethoxy-4-hydroxybenzaldehyde	C_9_H_10_O_3_	166	4.38
32.59	3,5-Di-*tert*-butylphenol	C_14_H_22_O	206	0.08
32.71	2-*Tert*-butyl-4-methoxyphenol	C_11_H_16_O_2_	180	0.05
32.85	3-[*N*,*N*-Dimethyl-*N*-(dodecyl)ammonio]propane-1-sulfonate	C_17_H_37_NO_3_S	335	0.33
37.38	2-(3-Acetyloxy-4,4,14-trimethylandrost-8-en-17 yl)propanoic acid	C_27_H_42_O_4_	430	0.20
37.88	(5α)-17-(2-Hydroxyethylidene)-17-ethylenedioxy-5α-cholestane	C_29_H_50_O_2_	430	0.06
39.52	(*Z*)-1-Chlorooctadec-9-ene	C_18_H_35_Cl	286	0.12
41.09	Phenylmethyl benzoate	C_14_H_12_O_2_	212	79.99
44.41	5-Amino-2-(4-methoxyphenyl)-2-methyl-2*H*-[1,2,4]triazolo[1,5-*a*][1,3,5]triazine	C_12_H_14_N_6_O	258	2.90
45.68	6-(4-Methylcyclohex-3-en-1-yl)hexan-2-one	C_18_H_26_O	258	0.22
47.5	Methyl hexadecanoate	C_17_H_34_O_2_	270	1.00
49.14	(3-Bromo-2,6,6-trimethylcyclohex-1-en-1-yl)(phenyl)methanone	C_16_H_19_BrO	306	0.38
49.7	Ethyl hexadecanoate	C_18_H_36_O_2_	284	0.42
51.03	[(2-Fluorophenyl)methyl]-9*H*-purin-6-amine	C_12_H_10_FN_5_	243	0.26
52.46	Methyl 4,6-*O*-benzylidene-α-d-hexopyranoside	C_14_H_18_O_6_	282	0.19
52.76	Methyl (*Z*)-octadec-9-enoate	C_19_H_36_O_2_	296	1.05
52.96	(3β,5*Z*)-3,17-Dihydroxypregn-5-en-20-one	C_21_H_32_O_3_	332	0.15
53.1	Methyl octadeca-10,13-diynoate	C_19_H_30_O_2_	290	0.11
53.75	Methyl octadecanoate	C_19_H_38_O_2_	298	0.81
54.98	Ethyl (*Z*)-octadec-9-enoate	C_20_H_38_O_2_	310	0.41
56.64	3′,4′,7-Trimethoxy-5-hydroxy-2-phenyl-4*H*-chromen-4-one	C_18_H_16_O_7_	344	0.14
60.88	6-Methoxy-2-phenyl-hexahydro-pyrano[3,2-*d*][1,3]dioxine-7,8-diol	C_14_H_18_O_6_	282	0.03
61.43	[(2-Fluorophenyl)methyl]-9*H*-purin-6-amine	C_12_H_10_FN_5_	243	0.36
62.61	(2*R*)-2-Phenyl-1,3-dioxolan-4-ylmethyl hexadecanoate	C_26_H_42_O_4_	418	0.18
63.33	6,8-Di-C-β-d-glucopyranosyl-5,7,3′,4′-tetrahydroxyflavone	C_27_H_30_O_16_	610	0.14
67.44	(3α,5α)-3,14-Dihydroxybufa-20,22-dienolide	C_24_H_34_O_4_	386	0.05
68.99	(3α,5α,14α,20α,22α,25*R*)-3-Hydroxy-11-oxospirost-8-en-11-one	C_27_H_40_O_4_	428	0.11
72.38	(*Z*)-Docos-13-enamide	C_22_H_43_NO	337	0.51
74.32	12-Hydroxy-2,2,8,8-tetramethyl-13-(3-methylbutanoyl)-1,7-dioxadispiro[4.0.5.3]tetradec-12-ene-11,14-dione	C_21_H_30_O_6_	378	0.22
76.29	1,2,7,8-Tetrahydro-ψ,ψ-carotene-1-ol	C_40_H_58_O	554	0.18
78.8	5,7-Dihydroxy-4′-hydroxy-flavone 5,7-di-*O*-β-d-glucopyranoside	C_27_H_30_O_15_	594	0.05
79	1,1′-Dimethoxy-1,1′,2,2′-tetrahydro-ψ,ψ-carotene	C_42_H_64_O_2_	600	0.11
81.75	3,3′-Dihydroxy-β,β-carotene-4,4′-dione	C_40_H_52_O_4_	596	0.03
85.14	(2*E*)-3,7,11,15-Tetramethylhexadec-2-en-1-yl (9*Z*,12*Z*)-octadeca-9,12-dienoate	C_38_H_70_O_2_	558	3.61

#### 3.2.3. HPLC

**Fig. 4:**
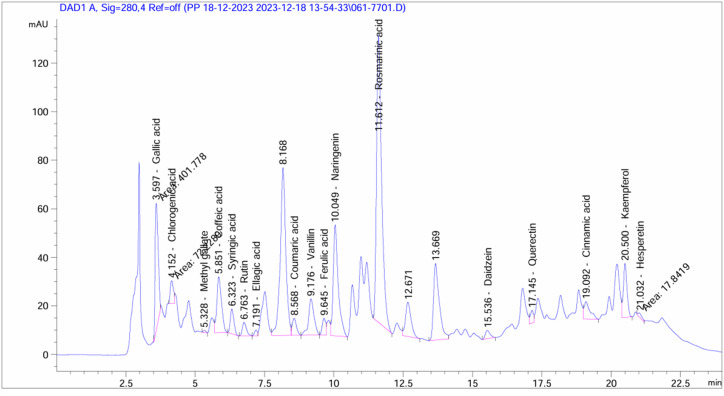
HPLC-based phytochemical profiling of *A. oryzae* NGM91 extract.

The HPLC chromatogram of the fungal crude extract revealed 17 well-resolved peaks, corresponding to the 17 phytochemical compounds listed in the accompanying results table. The chromatogram indicated good separation and resolution of the sample components as evidenced by the symmetrical, non-overlapping peak shapes. The peak areas quantified in the table indicate rosmarinic acid is the most abundant compound at 162.15 μg ml^−1^ (8107.61 μg g^−1^), corresponding to its large peak size and 54.2% relative abundance based on the total chromatogram peak area integration. Gallic acid is the second most abundant compound, comprising 14.4% of the sample with a concentration of 35.54 μg ml^−1^. Other major components based on peak area include vanillin (7.58 μg ml^−1^, 3.8% abundance), naringenin (52.31 μg ml^−1^, 2615.60 μg g^−1^, 20.5% abundance), and kaempferol (12.70 μg ml^−1^, 635.18 μg g^−1^, 7.2% abundance). The concentrations range from 0.35 μg ml^−1^ (methyl gallate) to 162.15 μg ml^−1^ (rosmarinic acid), exhibiting a wide variation in phytochemical composition (Fig. 4) (Table 3).

**Table 3:** Measured concentrations of bioactive compounds in the fungal crude extract

*A. oryzae* NGM91 crude extract			
	Area	Conc. (μg ml^−1^)	Conc. (μg g^−1^)
Gallic acid	401.78	35.54	1776.92
Chlorogenic acid	72.23	9.37	468.61
Methyl gallate	6.89	0.35	17.35
Caffeic acid	270.44	20.93	1046.41
Syringic acid	91.52	6.69	334.65
Rutin	57.64	8.50	425.12
Ellagic acid	16.92	1.69	84.49
Coumaric acid	74.81	2.66	133.11
Vanillin	203.98	7.58	379.02
Ferulic acid	61.33	3.56	178.14
Naringenin	572.32	52.31	2615.60
Rosmarinic acid	1512.36	162.15	8107.61
Daidzein	41.30	2.32	115.82
Quercetin	42.23	5.70	285.00
Cinnamic acid	105.01	1.88	94.02
Kaempferol	201.39	12.70	635.18
Hesperetin	17.84	0.88	43.86

### 3.3. Antioxidant assessment

**Fig. 5:**
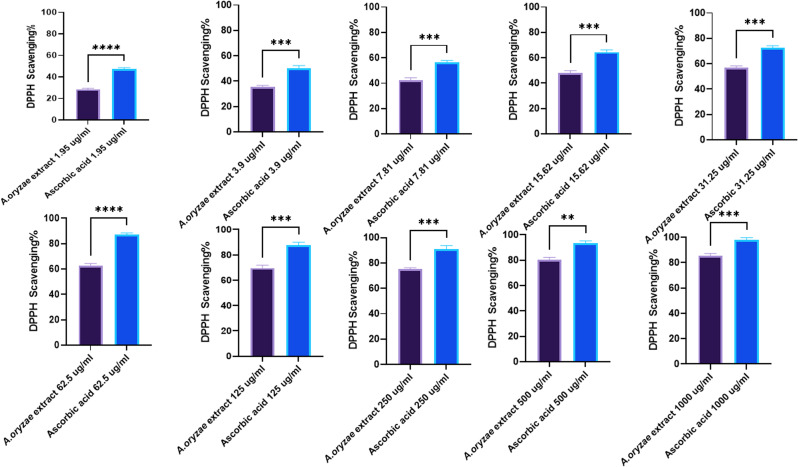
DPPH scavenging activity% of *A. oryzae* crude extract at varying concentrations (1.95–1000 μg ml^−1^) compared with ascorbic acid standard. Mean ± SD represents all data values. **p ≤ 0.01, ***p ≤ 0.001, ****p ≤ 0.0001.

The fungal extract was tested at 10 different concentrations ranging from 1.95 to 1000 μg ml^−1^. Each concentration was tested in triplicate, and the mean percent of DPPH scavenging was calculated. The results showed a dose-dependent increase in DPPH antioxidant activity with increasing the extract concentration. The IC_50_ value was determined to be 17.26 μg ml^−1^ compared to ascorbic acid IC_50_ of 2.85 μg ml^−1^. At the lowest concentration tested of 1.95 μg ml^−1^, the mean percent scavenging was 28.4%, increasing to 85% scavenging at the highest concentration of 1000 μg ml^−1^ (Fig. 5).

**Fig. 6:**
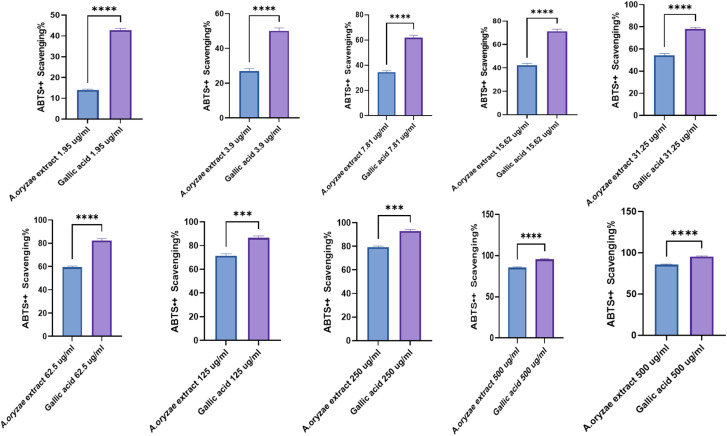
Dose-dependent antioxidant effects of *A. oryzae* NGM91 extract *vs.* gallic acid using ABTS˙^+^ assay (1.95–1000 μg ml^−1^). Mean ± SD represents all data values. **p ≤ 0.01, ***p ≤ 0.001, ****p ≤ 0.0001.

In ABTS˙^+^ testing, the lowest concentration tested of 1.95 μg ml^−1^ exhibited only minimal ABTS˙^+^ scavenging ability at 13.8%. The 500 μg per ml concentration yielded a considerable 85.7% ABTS˙^+^ radical scavenging. While the highest concentration of 1000 μg ml^−1^ displayed the maximum scavenging potential, nearing 90% at 89.6% (Fig. 6). Its IC_50_ value was calculated to be 27.91 μg ml^−1^ compared to 2.54 of gallic acid. Several moderate scavenging effects were observed with mid-range concentrations. For instance, 15.62 μg ml^−1^ resulted in 42.7% scavenging, while a higher 125 μg ml^−1^ concentration showed improved 71% activity.

The data presented in Table 4 demonstrates that the crude fungal extract possesses antioxidant capacity as measured by both (TAC) and (FRAP) assays. TAC evaluates the overall antioxidant power, while FRAP determines explicitly the ability to reduce ferric iron. The fungal extract exhibited considerable antioxidant activity in the TAC assay, with a mean value of 476.57 ± 0.68 μg per mg ascorbic acid equivalents (AAE). This high TAC activity reflects the sample's significant capacity to act as an antioxidant. Additionally, the extract showed antioxidant effects in the FRAP assay at a mean value of 302.62 ± 3.59 μg per mg AAE.

**Table 4:** TAC and FRAP values of *A. oryzae* NGM91 extract. Values expressed as mean ± SD μg mg^−1^

*A. oryzae* crude extract (AAE) μg mg^−1^	TAC (AAE) μg mg^−1^	FRAP (AAE) μg mg^−1^
	476.57 ± 0.68	302.62 ± 3.59

Based on the comprehensive chemical profiling and antioxidant analyses, the metabolites from *Aspergillus oryzae* NGM91 demonstrate substantial antioxidant capacity through multiple mechanisms. The FT-IR spectroscopy revealed the presence of phenolic compounds, evidenced by the broad O–H stretch at 3423 cm^−1^ (Table 1), constituting a primary structural feature responsible for free radical scavenging. The HPLC analysis particularly illuminated the abundance of potent antioxidant compounds, with rosmarinic acid emerging as the predominant constituent (162.15 μg ml^−1^), followed by significant concentrations of gallic acid (35.54 μg ml^−1^) and naringenin (52.31 μg ml^−1^) (Table 3). These phenolic acids and flavonoids possess multiple hydroxyl groups, facilitating electron donation to neutralize free radicals. The GC-MS profile further substantiated the presence of antioxidant-active compounds, notably ethyl vanillin (4.38%) and various phenolic derivatives (Table 2).

The considerable total antioxidant capacity (476.57 ± 0.68 μg per mg AAE) and ferric reducing power (302.62 ± 3.59 μg per mg AAE) (Table 4) correlate directly with the identified phenolic and flavonoid compounds. This synergistic interaction between rosmarinic acid, gallic acid, naringenin, and other identified phenolic compounds establishes the mechanistic basis for the extract's potent antioxidant properties through electron donation and radical neutralization pathways.^56,57^

### 3.4. Comparative COX isozyme inhibition profile of the anti-inflammatory fungal extract

**Fig. 7:**
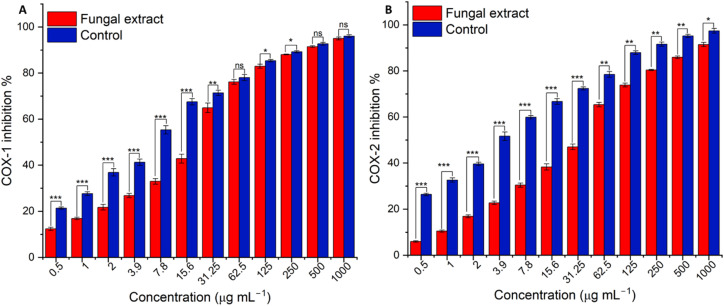
Comparative assessment of COX-1 (A) and COX-2 (B) enzyme inhibition: *A. oryzae* NGM91 extract (0.5–1000 μg ml^−1^) *vs.* celecoxib reference drug. Mean ± SD represents all data values. ns = non-significant, *p ≤ 0.1, **p ≤ 0.01, ***p ≤ 0.001.

The COX-1 inhibition assay results showed a concentration-dependent increase in inhibition percentage, ranging from 12.37% at 0.5 μg ml^−1^ to 95.03% at 1000 μg ml^−1^ of the extract. The IC_50_ value was determined to be 20.66 ± 0.74 μg ml^−1^ compared to 6.32 ± 0.74 μg ml^−1^ of celecoxib, indicating moderate potency. At lower concentrations of 0.5 to 15.6 μg ml^−1^, the crude extract exhibited mild COX-1 inhibition between 12.37% and 42.83%. However, at higher concentrations of 31.25 μg ml^−1^ and above, there was a sharp increase in COX-1 inhibition, reaching maximal inhibition of 95.03% at the highest concentration of 1000 μg ml^−1^ (Fig. 7).

Similarly, the COX-2 inhibition assay displayed a concentration-dependent response, with inhibition percentages increasing from 5.98% at 0.5 μg ml^−1^ to 91.47% at 1000 μg ml^−1^ of the crude extract. The IC_50_ was calculated as 36.32 ± 1.24 μg ml^−1^ compared to 3.73 ± 0.29 μg ml^−1^ of celecoxib. At lower concentrations between 0.5 and 15.6 μg ml^−1^, the fungal extract showed minimal COX-2 inhibition ranging from 5.98% to 38.29%. At higher concentrations starting from 31.25 μg ml^−1^, there was a steep increase in COX-2 inhibition, approaching maximum 91.47% inhibition at 1000 μg ml^−1^ (Fig. 7).

### 3.5. *A. oryzae* NGM91 extract's cancer cell cytotoxicity

**Fig. 8:**
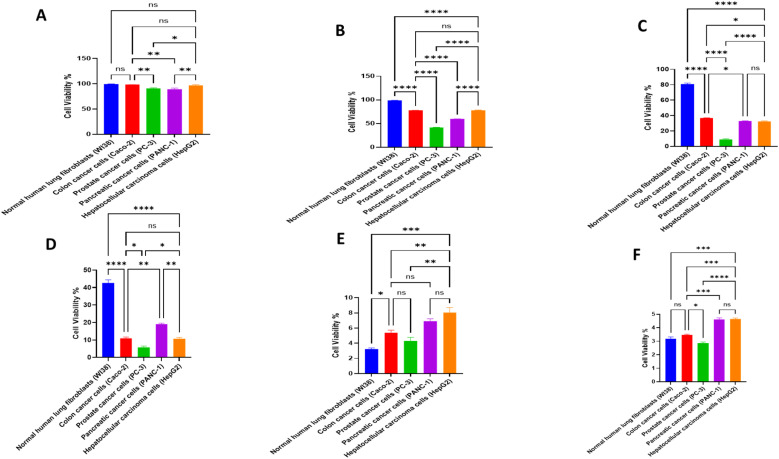
Comparative cytotoxicity assessment of different concentrations of *A. oryzae* NGM91 extract against four cancer cell lines and normal lung fibroblasts (A = 31.2 μg ml^−1^, B = 62.5 μg ml^−1^, C = 125 μg ml^−1^, D = 250 μg ml^−1^, E = 500 μg ml^−1^, F = 1000 μg ml^−1^). Mean ± SD represents all data values. ns = non-significant, *p ≤ 0.1, **p ≤ 0.01, ***p ≤ 0.001, ****p ≤ 0.0001.

**Fig. 9:**
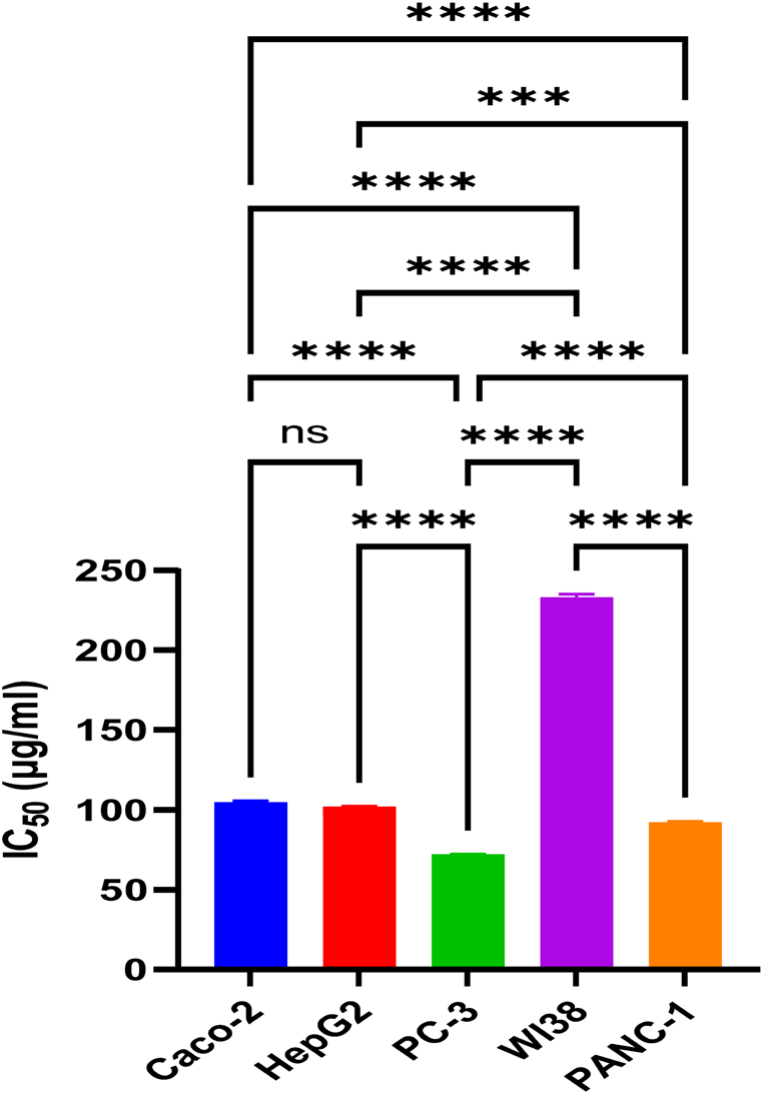
IC_50_ concentrations of *A. oryzae* NGM91 extract inhibiting 50% viability of each cell line. Data are presented as mean ± SD.

Based on morphological assessment, untreated WI-38 lung fibroblasts displayed normal elongated, spindle-shaped morphology with intact cell membranes. At the lowest tested concentrations 31.25–250 μg ml^−1^ of the fungal extract, cell morphology remained largely normal and similar to that of untreated control cells, with most cells exhibiting their typical elongated, spindle shape and intact cell membranes (Fig. S1†). Where at its highest tested concentrations, rounder, shrunken cells with granular debris formation. While cytotoxic quantitative assessment revealed at 1000, viability was 3.33%. However, viability increased to 43.89% at 250 μg ml^−1^, 81.82% at 125 μg ml^−1^, 98.69% at 62.5 μg ml^−1^, and 99.8% at 31.25 μg ml^−1^, showing improved viability at lower concentrations (Fig. 8). The IC_50_ was 230.31 ± 6.29 μg ml^−1^, confirming increased viability percentages with decreasing concentrations (Fig. 9).

Concerning the colon cancer (Caco-2) cell line, untreated Caco-2 colon cancer cells displayed typical epithelial morphology, with a polygonal shape and clear, defined cell edges. After treatment with varying extract concentrations, concentration-dependent morphological changes were observed. Cells were severely damaged at higher concentrations with complete loss of normal morphology. Nearly all cells appeared rounded and shrunken, with blurred, indistinct edges. Cellular debris was visible, indicating cell disintegration (Fig. S2†). As the concentration decreased, cell morphology improved, with some polygonal-shaped cells visible alongside rounded cells and debris. At the lowest tested concentrations, cell morphology approached that of untreated cells, though some residual indicators of toxicity remained. The quantitative assessment revealed severe cytotoxicity at higher concentrations, with 3.5% and 5.6% viability at 1000 and 500 μg ml^−1^, respectively (Fig. 8). Viability rose in a concentration-dependent manner to 10.3% at 250 μg ml^−1^, 36.5% at 125 μg ml^−1^, 78.2% at 62.5 μg ml^−1^, and 98.3% at 31.25 μg ml^−1^ and the IC_50_ was 104.69 ± 1.67 μg ml^−1^ (Fig. 9).

Next, the cytotoxicity of *A. oryzae* extract was then tested against hepatocellular carcinoma cells (HepG2) at concentrations ranging from 1000 μg ml^−1^ to 31.25 μg ml^−1^. Untreated HepG2 cells appear healthy and normal in morphology. They are adhered to the plate surface and have a polygonal shape with defined cell borders. While treatment of HepG2 cells with the extract results in both concentration-dependent morphological changes, including cell shrinkage and rounding, as well as decreases in cell viability (Fig. S3†). The fungal crude extract was highly cytotoxic at the highest concentrations of 1000 μg ml^−1^ and 500 μg ml^−1^, reducing cell viability to only 4.7% and 7.5%, respectively (Fig. 8). Toxicity decreased at 250 μg ml^−1^ (viability 10.3%) and further dropped at 125 μg ml^−1^ (viability 31.9%). Much lower toxicity was observed at 62.5 μg ml^−1^ (viability 78.8%) and 31.25 μg ml^−1^ (viability 97.8%), its IC_50_ value was calculated to be 100.36 ± 1.76 μg ml^−1^ (Fig. 9).

Treatment of prostate cancer (PC3) cells with the fungal extract induced both concentration-dependent cytotoxicity, with an IC_50_ of 70.47 ± 0.26 μg ml^−1^ (Fig. 9), as well as morphological alterations ranging from cell shrinkage and rounding at low concentrations to near-complete cell death and detachment at the highest concentrations. The untreated PC3 cells appear healthy and normal, adhered to the plate surface with defined cell borders. After treatment with crude extract, a concentration-dependent cytotoxic effect was observed. At 1000 μg ml^−1^ of the extract, only 2.9% of PC3 cells remain viable. Viability increases slightly to 4.6% and 6.3% at 500 and 250 μg ml^−1^ of the fungal extract, respectively. At the lower concentrations of 125, 62.5, and 31.25 μg ml^−1^, viability continues to improve, ranging from 9.5% to 91.6% (Fig. 8). Morphologically, at the highest extract concentrations of 1000 and 500 μg ml^−1^, nearly all PC3 cells have died and detached from the plate. At 250 μg ml^−1^, there is also significant cell death and loss of adherence. As the concentration decreases, more cells remain attached, but they appear less confluent and smaller compared to untreated cells (Fig. S4†). At the lowest concentrations of 62.5 and 31.25 μg ml^−1^, morphological changes are more subtle, with some rounding up, but most cells remain attached and viable.

Finally, for the pancreatic cancer (PANC-1) cell line, microscope images reveal noticeable morphological differences between control, untreated, and treated PANC-1 cells. The untreated PANC-1 cells appear healthy with normal morphology, cells are well spread with defined edges and retain their epithelial shape. In contrast, the crude extract treated PANC-1 cells exhibit progressive morphological anomalies and deterioration correlated with increasing extract concentration. At lower concentrations, like 31.25 μg ml^−1^, cells start rounding up and shrinking, with some floating dead cells visible. Higher concentrations of 250 and 500 μg ml^−1^ induce more extensive cytotoxic effects, with most cells severely shrunken and detached, indicating widespread cell death. Ultimately, at the highest tested concentration of 1000 μg ml^−1^, the fungal extract results in complete death of PANC-1 cell morphology with predominantly cell debris present (Fig. S5†).

Quantitative cytotoxicity assays demonstrate the promising *in vitro* anticancer effects of the fungal extract against PANC-1 cells across a wide dosage range from 31.25 to 1000 μg ml^−1^. At its highest tested concentration of 1000 μg ml^−1^, the crude extract exhibited potent toxicity with only 4.7% cell viability and 95.3% inhibition (Fig. 8). Appreciable toxicity of 93.4% was retained even at a lower 500 μg per ml concentration. Notably, even at the lowest tested dose of 31.25 μg ml^−1^, the extract maintained considerable cytotoxicity of 9.1% against PANC-1 cells, and its IC_50_ value quantitatively was calculated as 90.42 ± 0.87 μg ml^−1^ (Fig. 9).

The selective cytotoxicity profile of the explored fungal metabolite emerges from the synergistic actions of multiple compounds rather than the effect of a single constituent. The FT-IR spectroscopic profile, particularly the strong O–H stretch at 3423 cm^−1^ and carbonyl stretch at 1647 cm^−1^, indicates a rich presence of phenolic and carbonyl-containing compounds. These functional groups, coupled with the amine signatures (N–H bend at 1541 cm^−1^) (Table 1), suggest structures capable of hydrogen bonding and electron donation – properties crucial for interaction with cellular targets. The high concentration of rosmarinic acid (8107.61 μg g^−1^) is particularly significant given its documented abilities in reactive oxygen species modulation and inflammatory pathway regulation.^58^ The substantial presence of naringenin (2615.60 μg g^−1^) and gallic acid (1776.92 μg g^−1^) (Fig. 4) suggests multiple mechanisms of action, including potential epigenetic modulation and cell cycle regulation.^59,60^

The presence of multiple phenolic compounds (chlorogenic acid: 468.61 μg g^−1^, caffeic acid: 1046.41 μg g^−1^) alongside flavonoids (kaempferol: 635.18 μg g^−1^, quercetin: 285 μg g^−1^) indicates potential synergistic interactions. This phytochemical profile directly correlates with the extract's high antioxidant capacity (TAC: 476.57 ± 0.68 μg per mg AAE; FRAP: 302.62 ± 3.59 μg per mg AAE) and impressive IC_50_ values in DPPH (17.26 μg ml^−1^) and ABTS (27.91 μg ml^−1^) assays. These compounds traditionally act through distinct but complementary pathways, from direct antioxidant activities to modulation of cell signalling cascades and apoptotic pathways.^61–63^ The hydroxyl groups in these polyphenols facilitate electron donation for radical neutralization, explaining both their antioxidant properties and paradoxical pro-oxidant effects in cancer cells (evidenced by increased MDA and NO in treated PC3 cells). Such structure–activity relationship becomes apparent when examining the FT-IR data (O–H stretch at 3423 cm^−1^), confirming phenolic structures capable of redox modulation.

The GC-MS analysis revealed benzyl benzoate as the dominant compound (79.99%), which merits attention given its potential role in membrane permeability modulation.^64^ Also, ethyl vanillin (4.38%) and phytyl linoleate (3.61%) hint synergistic effects. Minor constituents like 3,17-dihydroxypregn-5-en-20-one (0.15%) (Table 2) and flavonoid derivatives may also contribute to the anticancer activity despite their lower concentrations (Ullah *et al.*, 2020; Zhang *et al.*, 2019). The concentration-dependent morphological changes observed in cancer cells (Fig. S1–S5†), particularly the progression from cell shrinkage to complete membrane disruption, suggest a multi-targeted approach rather than a single mechanism.^65,66^

### 3.6. Oxidative stress damage assessment in cancerous cell lines

**Fig. 10:**
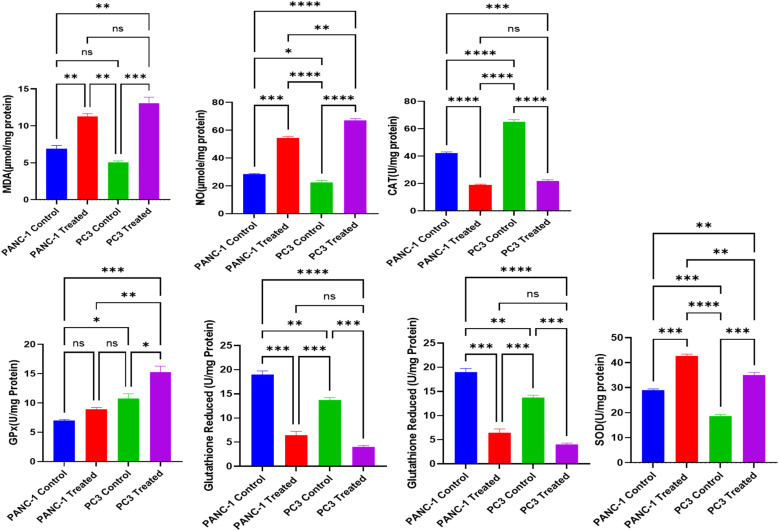
Cellular stress response in PANC-1 and PC3 cells: control *vs. A. oryzae* NGM91 extract treatment. Mean ± SD represents all data values. *p ≤ 0.1, **p ≤ 0.01, ***p ≤ 0.001, ****p ≤ 0.0001, ns = non-significant.

Based on the cytotoxicity results, where the lowest IC_50s_ given were IC_50_ of 70.47 ± 0.26 μg ml^−1^ for PC3 and 90.42 ± 0.87 μg ml^−1^ for PANC-1, therefore we stepped forward to assess the oxidative stress damage. For PANC-1 cells, treatment with *A. oryzae* NGM91 extract induced significant increases in MDA levels from 6.57 ± 0.37 to 10.95 ± 0.15 μmol per mg protein, and NO levels elevated from 27.93 ± 0.35 to 53.50 ± 1.31 μmole per mg protein. The antioxidant enzyme activities showed marked changes, with catalase decreasing from 41.93 ± 1.80 to 18.37 ± 0.76 U per mg protein, glutathione peroxidase increasing from 6.89 ± 0.36 to 8.76 ± 0.58 U per mg protein, reduced glutathione declining from 18.56 ± 0.23 to 7.00 ± 0.15 U per mg protein, and SOD levels rising from 29.37 ± 0.71 to 42.07 ± 1.38 U per mg protein (Fig. 10).

For PC3 cells, the extract triggered more pronounced oxidative stress responses. MDA levels increased from 5.20 ± 0.26 to 13.63 ± 0.40 μmol per mg protein, while NO levels surged from 21.43 ± 1.22 to 66.13 ± 5.87 μmole per mg protein. The antioxidant defense markers showed substantial alterations, with catalase decreasing from 64.07 ± 1.11 to 21.17 ± 1.35 U per mg protein, glutathione peroxidase increasing from 10.18 ± 0.23 to 14.47 ± 0.71 U per mg protein, reduced glutathione dropping from 13.45 ± 1.10 to 4.23 ± 0.26 U per mg protein, and SOD levels elevating from 18.10 ± 0.36 to 34.47 ± 0.80 U per mg protein (Fig. 10).

### 3.7. Gene expression, comet assay, DNA fragmentation, and cell cycle arrest in treated PC3 cells

**Fig. 11:**
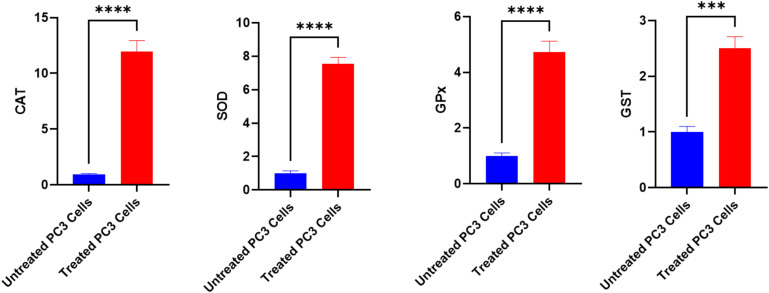
The alterations of CAT, SOD, GPx and GST expression in untreated and treated PC3 cells with the fungal extract. Mean ± SD represents all data values. ***p ≤ 0.001, ****p ≤ 0.0001.

The study revealed a complex interplay between oxidative stress responses and cellular outcomes in treated PC3 cells. Starting with antioxidant gene expression, the antioxidant enzyme genes (CAT, SOD, GPx, and GST) were significantly downregulated (*P* < 0.01) in untreated prostate cancer cells compared to treated cells. Conversely, these genes were overexpressed considerably (*P* < 0.01) in treated PC3 cells *versus* untreated controls. Treated PC3 cells exhibited remarkable upregulation of all measured antioxidant genes, with CAT showing the most dramatic increase (1195%), followed by SOD (788%), GPx (473%), and GST (251%). However, this transcriptional response did not translate directly to enzyme activity levels. While SOD and GPx activities showed modest increases (90% and 42% increases, respectively), catalase activity paradoxically decreased by 67%, suggesting potential post-transcriptional interference or direct enzyme inhibition by oxidative stress products (Fig. 11).

The oxidative stress markers, as shown in Fig. 10, support this interpretation, with MDA levels increasing by 162% (from 5.20 to 13.63 μmol per mg protein) and NO levels surging by 208% (from 21.43 to 66.13 μmole per mg protein). This substantial increase in oxidative stress markers, despite elevated antioxidant gene expression, indicates that the cells' defensive responses were overwhelmed. This oxidative burden is reflected in the significant reduction of reduced glutathione (68.5% decrease), a critical cellular antioxidant defensive reservoir.

**Fig. 12:**
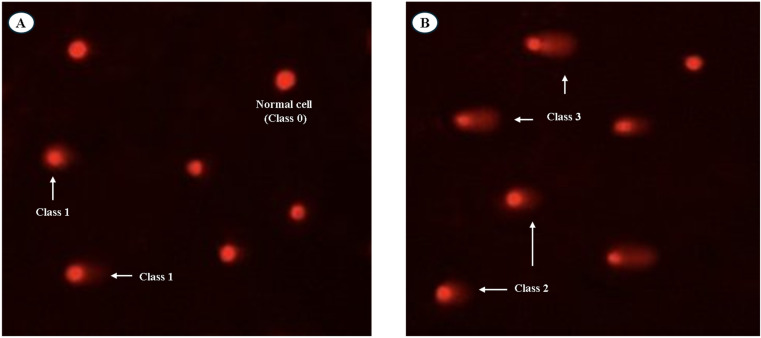
Visual scoring of DNA damage using comet assay in PC3 cells. (A) Image showing normal DNA (class 0) and damaged DNA (class 1). (B) Image showing higher levels of DNA damage (classes 2 and 3) in PC3 cells treated with the fungal extract. DNA fragmentation analysis further confirmed this early DNA damage. The results proved that negative control PC3 cell lines revealed a significant decrease (*P* < 0.01) in DNA fragmentation rates (13.6 ± 1.66) compared with those in treated samples. Nevertheless, the DNA fragmentation values were increased significantly (*P* < 0.01) in treated PC3 cell lines (27.9 ± 1.96) compared with negative control cancer cell lines (13.6 ± 1.66) (Table 6) (Fig. S6†).

The downstream effects of this oxidative assault are evident in the DNA damage analysis. The comet assay revealed that the untreated PC3 cancer cell line showed a significant decrease in DNA damage values (11.2 ± 0.85) in comparison with the treated PC3 (*P* < 0.05). In contrast, the DNA damage values were raised significantly in the treated PC3 cancer cell line sample (19.8 ± 2.50) compared with the negative control (11.2 ± 1.71) (*P* < 0.01) (Tables 5 and 6). In addition, the DNA damage in PC3 cancer cell lines showed a higher percentage of class 3 displaying high tail length migration than those in negative PC3 cell lines (Fig. 12).

**Table 5:** Visual score of DNA damage in PC3 cell line treated with *A. oryzae*'s extract

Treatment	No. of samples	No. of cells		Class^b^				DNA damaged cells% (mean ± SD)
		Analyzed^a^	Comets	0	1	2	3	
Untreated PC3 cells	4	600	67	533	29	23	15	11.2 ± 1.71^b^
Treated PC3 cells	4	600	119	481	43	37	39	19.8 ± 2.50^a^

a Number of cells examined per group.

b Class 0 = no tail; 1 = tail length < diameter of nucleus; 2 = tail length between 1× and 2× the diameter of nucleus; and 3 = tail length > 2× the diameter of nucleus. Means with different superscripts (a, b) between groups in the same treatment are significantly different at *P* < 0.05. Data are presented as mean ± SD.

**Table 6:** DNA fragmentation detected in negative and treated PC3 cell lines^a^

Treatment	DNA fragmentation% (mean ± SD)	% of control
PC3 cell line (−ve)	13.6 ± 1.66^b^	100.0
Treated PC3 cell line	27.9 ± 1.96^a^	205.1

a Means bearing distinct superscripts (a, b) between groups within identical columns indicate significant differences at *P* < 0.05.

**Fig. 13:**
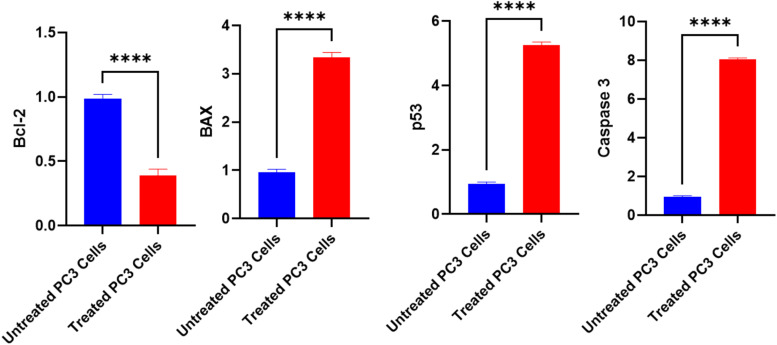
The alterations of BCL-2, BAX, P53, and Caspase-3 genes expression in untreated and treated PC3 cells with the fungal extract. Mean ± SEM expresses all data points. ***p ≤ 0.001, ****p ≤ 0.0001.

The cellular response to this damage is reflected in the expression patterns of apoptotic regulators. The anti-apoptotic BCL-2 showed a 61% decrease, while pro-apoptotic genes demonstrated substantial upregulation: BAX (343%), p53 (525%), and most notably, Caspase-3 (806%). These changes in expression ratios, particularly the BAX/BCL-2 ratio shift, strongly indicate the activation of apoptotic pathways (Fig. 13).

**Fig. 14:**
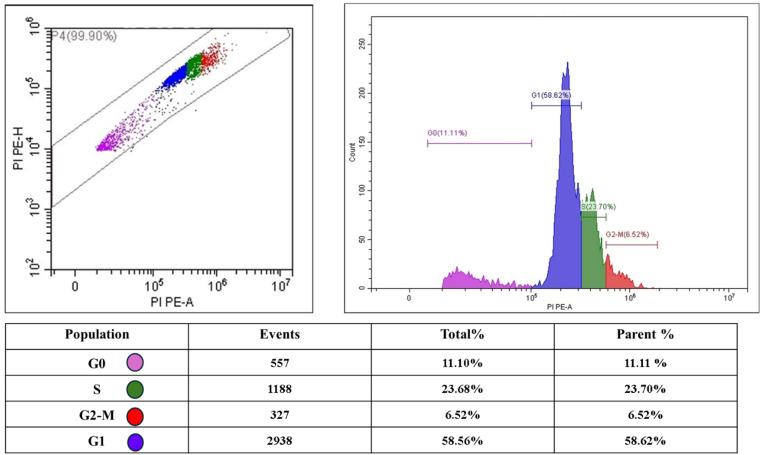
Cell cycle phase distribution of control PC3 cells demonstrating normal proliferation patterns.

Looking at the cell cycle analysis of untreated PC3 cells, the data revealed distinctive distribution patterns across different phases. Most cells (58.62%) were found in the G1 phase, representing the initial growth period. Following this, 23.70% of the cells were observed in the S phase, where DNA replication occurs. A smaller fraction of cells (11.11%) resided in the G0 phase, indicating a quiescent state. The G2-M phase, marking the preparation for and execution of cell division, contained the smallest population at 6.52% (Fig. 14). This distribution suggests that most PC3 cells in the untreated condition maintain normal proliferation patterns, predominantly occupying the G1 phase while actively progressing through the cell cycle stages.

**Fig. 15:**
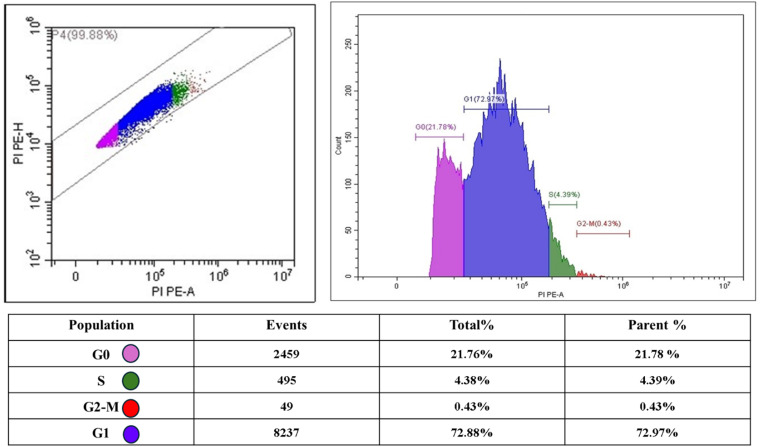
Quantitative analysis of cell cycle arrest in PC3 cells treated with *A. oryzae*'s extract, revealing G1 phase accumulation and diminished G2/M population.

On the other hand, the cell cycle analysis of PC3 cells treated with the fungal extract displayed a significant shift in phase distribution. The G1 phase shows a marked increase to 72.97% of the cell population, indicating substantial G1 arrest. Notably, the S phase fraction dropped dramatically to 4.39%, reflecting a strong reduction in DNA synthesis activity. The G2–M population decreased sharply to just 0.43%, demonstrating minimal cell division activity. Meanwhile, the G0 phase showed an elevation to 21.78%, suggesting that more cells entered a quiescent state (Fig. 15). These alterations in cell cycle distribution, particularly the pronounced G1 accumulation and diminished S phase population, strongly point to the fungal extract's antiproliferative effect on PC3 cells through G1 phase arrest mechanisms.

Integrating these findings reveals a clear mechanistic pathway. The initial molecular changes, evidenced by the dramatic upregulation of antioxidant genes, appear to be a cellular response to oxidative stress, as validated by the increased MDA and NO levels (Fig. 10). This oxidative stress environment, despite the compensatory increase in antioxidant gene expression, led to significant DNA damage, as demonstrated by both comet assay and DNA fragmentation analyses (Fig. S6†). The concurrent activation of the p53-dependent apoptotic pathway, shown by the substantial increases in P53, BAX, and Caspase-3 expression, along with the decrease in BCL-2, suggests that the extract triggers cell death through oxidative stress-mediated DNA damage (Fig. 13). This comprehensive data set demonstrated that *A. oryzae* NGM91 extract induces PC3 cancer cell death through a well-coordinated sequence of molecular and cellular events, making it a promising candidate for further anti-cancer research.

Future work should include *in vivo* studies to validate the anticancer effects, examine potential toxicity in animal models, and evaluate pharmacokinetic properties. Additional research could explore synergistic effects with existing cancer treatments, develop targeted delivery systems for the active compounds, and investigate the extract's potential against metastatic and drug-resistant cancers. Furthermore, isolating and characterizing individual bioactive compounds could lead to more potent and selective anticancer agents. Engineering studies for optimized production and standardization of the extract would be valuable for potential therapeutic development.

## 4 Conclusion

Based on the comprehensive analysis of *Aspergillus oryzae* NGM91 marine-derived extract, this study demonstrated significant anticancer potential through multiple mechanisms. Chemical profiling revealed a complex mixture dominated by benzyl benzoate (79.99%) and rosmarinic acid as major constituents, with the extract exhibiting potent antioxidant activity across DPPH, ABTS, TAC, and FRAP assays. The fungal extract showed selective cytotoxicity against cancer cell lines while maintaining relatively low toxicity toward normal WI-38 cells, with PC3 prostate cancer cells showing particular sensitivity (IC_50_ 70.47 μg ml^−1^). Mechanistic investigations in PC3 cells revealed that the extract induced oxidative stress, evidenced by elevated MDA (162% increase) and NO (208% increase) levels, despite the upregulation of antioxidant genes. This oxidative burden led to DNA fragmentation, confirmed by comet assay and gel electrophoresis, alongside significant modulation of apoptotic genes – downregulation of anti-apoptotic BCL-2 (61% decrease), and upregulation of pro-apoptotic BAX (343%), p53 (525%), and Caspase-3 (806%). Cell cycle analysis revealed pronounced G1 phase arrest (72.97%) with a concurrent reduction in S phase (4.39%) and G2–M phase (0.43%) populations. These findings establish *A. oryzae* NGM91 extract as a promising source of anticancer compounds that operate through oxidative stress-mediated DNA damage and apoptosis induction, warranting further investigation for potential therapeutic development.

## Data availability

The data supporting this article have been included as part of the ESI.†

## Author contributions

R. H. N.: methodology, investigation, and formal analysis. Z. F. A.: methodology, investigation, and validation. Z. A.: methodology, investigation, and data curation. A. A.: methodology, visualization, and data curation. T. G.: methodology, investigation, and data curation. A. M. A. S.: investigation and methodology. M. E.: methodology and formal analysis. H. A. A.: methodology and visualization. G. S. A.: data curation formal analysis and visualization. N. A. T.: conceptualization, supervision, and project administration. A. F.: conceptualization, writing – review & editing, and writing – original draft. Ahmed Ghareeb: conceptualization, methodology, investigation, supervision, writing – original draft and writing – review & editing.

## Conflicts of interest

The authors declare no competing interests.

## Supplementary Material

RA-015-D5RA02028J-s001
