# Supplementary material for: Oxidative stress-induced DNA damage and apoptosis in multiple cancer cell lines: novel anticancer properties of marine Aspergillus oryzae NGM91 extract
Source: RSC Adv. 2025 May 22;15(22):17203–21. doi: 10.1039/d5ra02028j (PMC12096281; doi:10.1039/d5ra02028j)
Supplement: RA-015-D5RA02028J-s001 [file RA-015-D5RA02028J-s001.pdf]

Supplementary Information (SI) for RSC Advances.
This journal is © The Royal Society of Chemistry 2025

Oxidative Stress-Induced DNA Damage and Apoptosis in Multiple Cancer Cell Lines: Novel Anticancer Properties of Marine *Aspergillus oryzae* NGM91 Extract

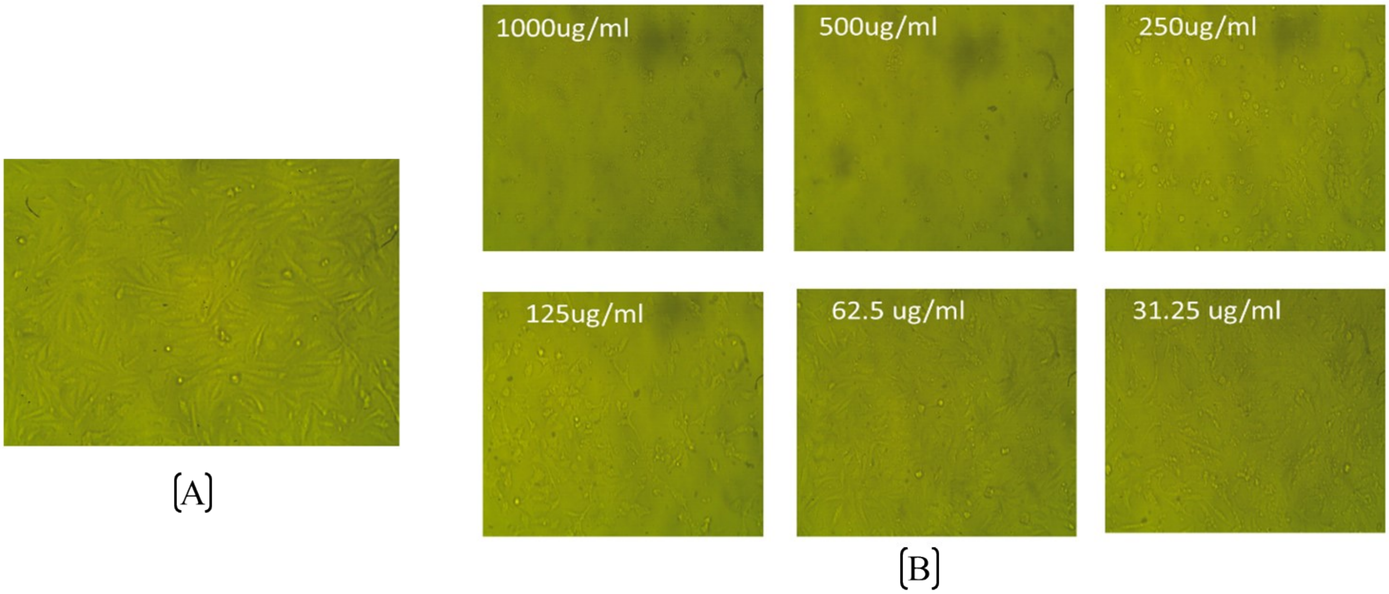

Figure S1. Morphology of WI-38 cells before and after *A. oryzae* NGM91 crude extract (A) Untreated control WI-38 cells (B) WI-38 cells treated with 31.25-1000 µg/mL fungal extract

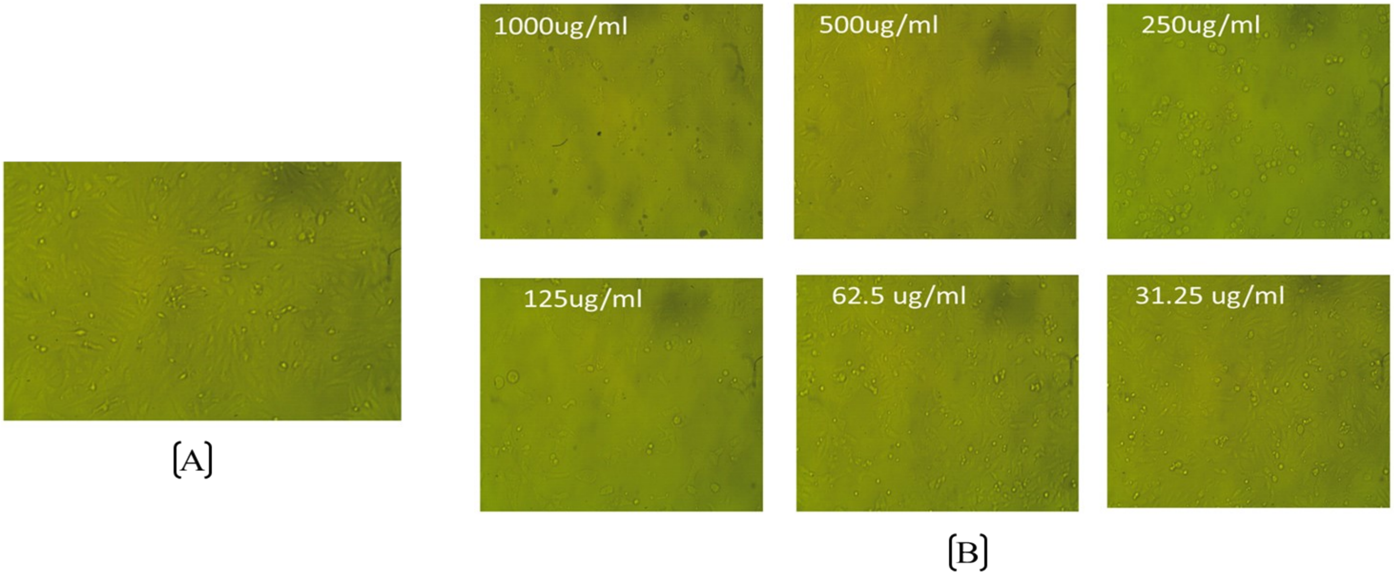

Figure S2. Morphology of Caco-2 cells before and after *A. oryzae* NGM91 crude extract treatment (A) Untreated control Caco-2 cells (B) Caco-2 cells treated with 31.25-1000 µg/mL fungal extract

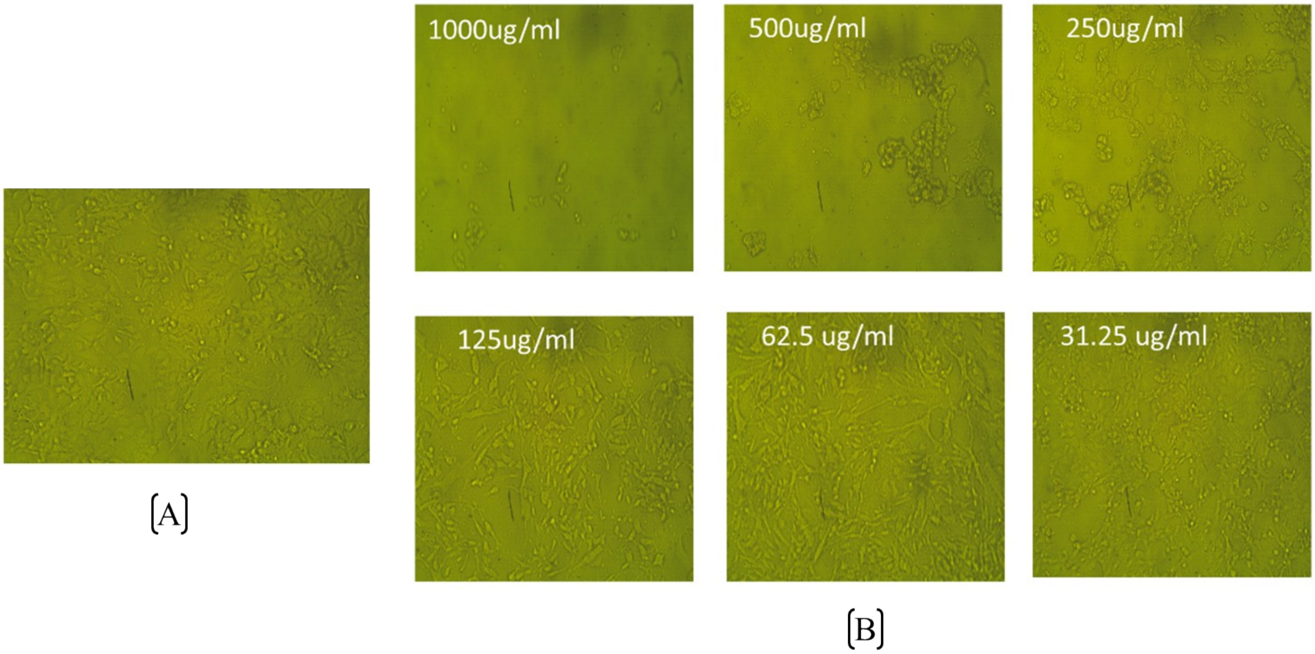

Figure S3. Morphology of HepG2 cells before and after *A. oryzae* NGM91 crude extract treatment (A) Untreated control HepG2 cells (B) HepG2 cells treated with 31.25-1000 µg/mL fungal extract

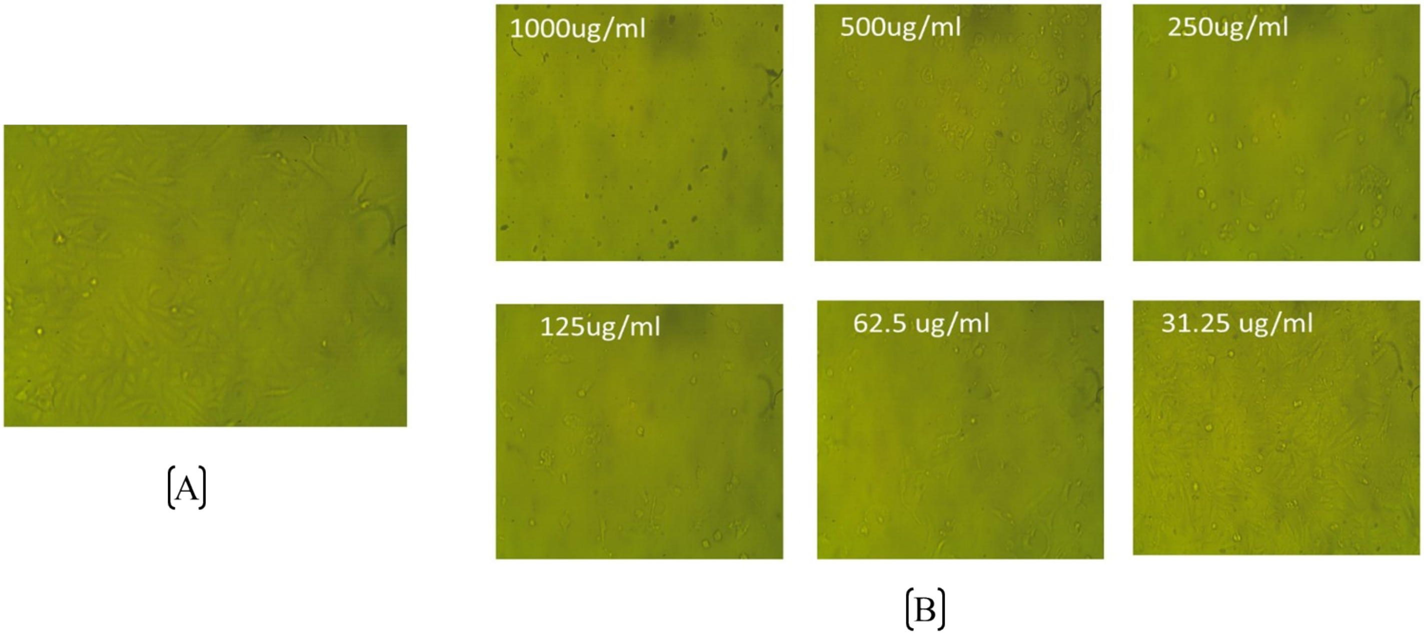

Figure S4. Morphology of Pc3 cells before and after *A. oryzae* NGM91 crude extract treatment (A) Untreated control Pc3 cells (B) Pc3 cells treated with 31.25-1000 μg/mL fungal extract

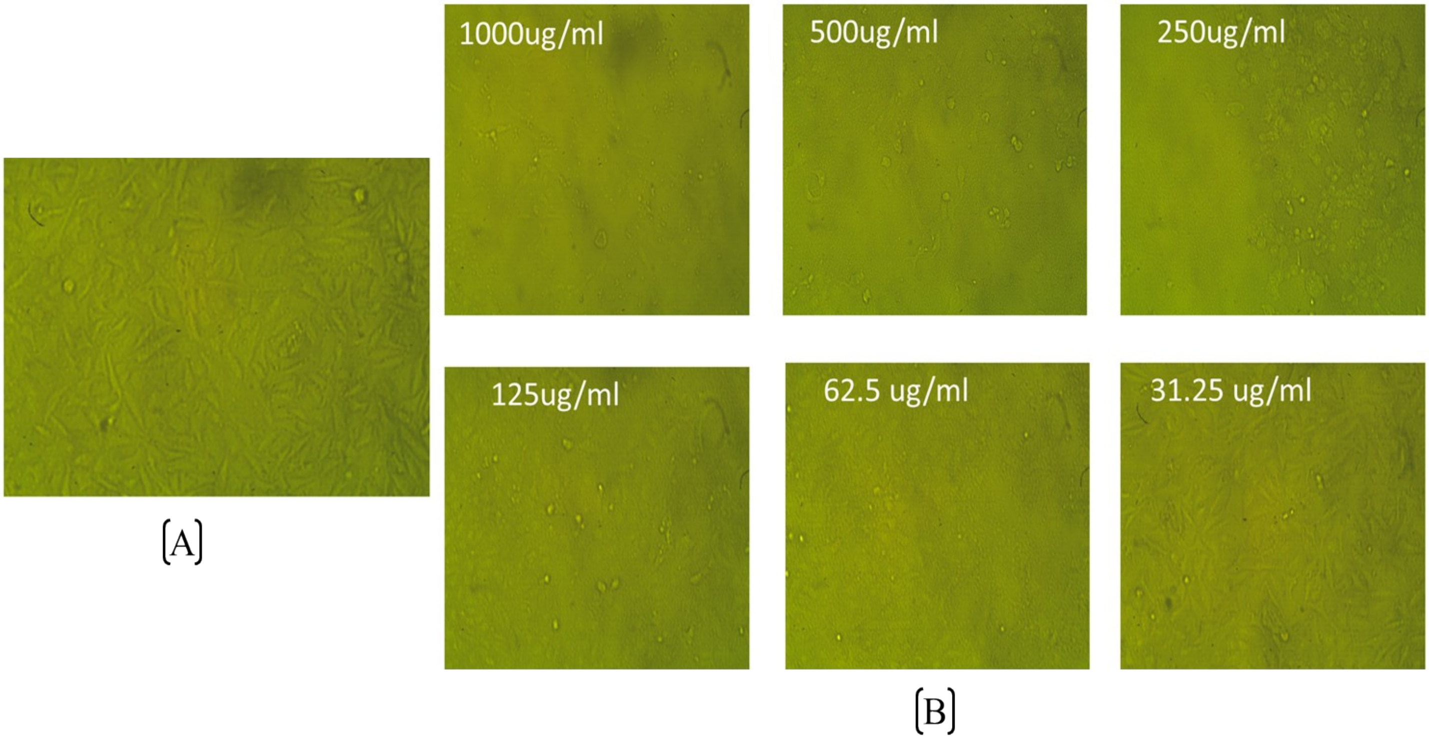

Figure S5. Morphology of PANC-1 cells before and after *A. oryzae* NGM91 crude extract treatment (A) Untreated control PANC-1 cells (B) PANC-1 cells treated with 31.25-1000 µg/mL fungal extract

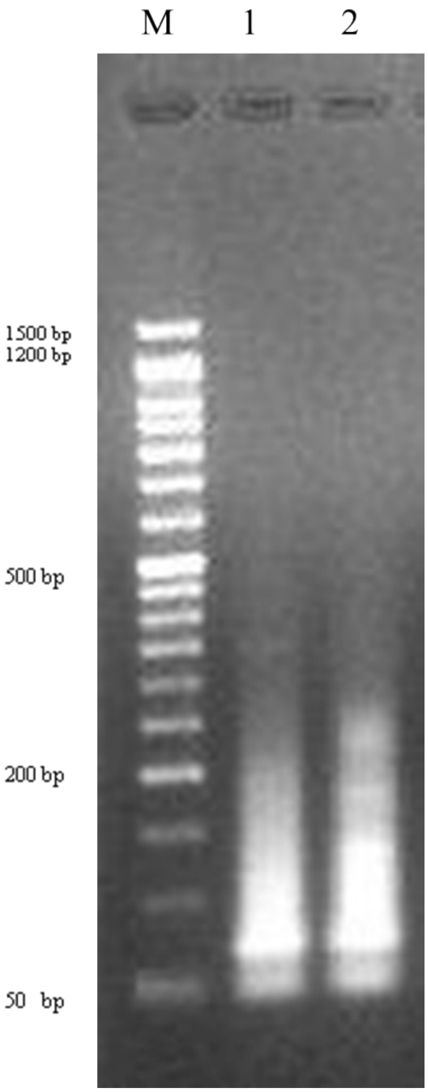

Figure S6. DNA fragmentation detected with Agarose gel in negative and treated prostate cancer cell lines. M: represents DNA marker, Lane 1: represents negative control of prostate cancer cell lines, Lane 2: represents treated prostate cancer cell lines.

Table S1: Primer's sequence used for *qRT-PCR* of PC3 cell lines

Gene	Primer sequence	GenBank (accession no)
**CAT**	F`: GAG CCT ACG TCC TGA GTC TC	NM_001752.4

	R`: ATC CCG GAT GCC ATA GTC AG	
**SOD**	F`: GGA GAC TTG GGC AAT GTG AC<br>R`: CAC AAG CCA AAC GAC TTC CA	AY450286.1
**GPX**	F`: TAA TGG AGC TGC ACT GGG AA<br>R`: CCT AAA GGG CAA GGG GAG AA	NM_001008397.4
**GST**	F`: TGA TGT CCT TGA CCT CCA CC<br>R`: ATC TGA GCA TCC CTT AGC CC	M99422.1
**Bcl-2**	F`: GCC TTC TTT GAG TTC GGT GG<br>R`: GAA ATC AAA CAG AGG CCG CA	EU287875.1
**BAX**	F`: AAG AAG CTG AGC GAG TGT CT<br>R`: GTT CTG ATC AGT TCC GGC AC	L22473.1
**P53**	F`: CAT CTG GCC TTG AAA CCA CC<br>R`: TGG GAT GGG GTG AGA TTT CC	AB082923.1
**Caspase-3**	F`: ACT GGA CTG TGG CAT TGA GA<br>R`: GCA CAA AGC GAC TGG ATG AA	NM_004346.4
**GAPDH**	F`: CCA AGG AGT AAG ACC CCT GG<br>R`: TGG TTG AGC ACA GGG TAC TT	NM_001256799.3

CAT: Catalase gene; SOD: Superoxide dismutase gene; GPx: Glutathione peroxidase gene; GST: Glutathione transferase gene; Bcl-2: B-cell lymphoma-2 gene; BAX: Bcl-2-associated X protein encoding gene; p53: tumor suppressor gene; GAPDH: Glyceraldehyde-3-phosphate dehydrogenase
